# Transcriptome analysis of coding and long non-coding RNAs highlights the regulatory network of cascade initiation of permanent molars in miniature pigs

**DOI:** 10.1186/s12864-017-3546-4

**Published:** 2017-02-10

**Authors:** Fu Wang, Yang Li, Xiaoshan Wu, Min Yang, Wei Cong, Zhipeng Fan, Jinsong Wang, Chunmei Zhang, Jie Du, Songlin Wang

**Affiliations:** 1 Molecular Laboratory for Gene Therapy & Tooth Regeneration, Beijing Key Laboratory of Tooth Regeneration and Function Reconstruction, School of Stomatology, Capital Medical University, Beijing, 100050 China; 2 Department of Oral Basic Science, School of Stomatology, Dalian Medical University, Liaoning, 116044 China; 3 Laboratory of Molecular Signaling and Stem Cells Therapy, Beijing Key Laboratory of Tooth Regeneration and Function Reconstruction, School of Stomatology, Capital Medical University, Beijing, 100050 China; 4 Department of Biochemistry and Molecular Biology, School of Basic Medical Sciences, Capital Medical University, Beijing, 100069 China; 5 Department of Physiology and Pathophysiology, Beijing AnZhen Hospital the Key Laboratory of Remodeling-Related Cardiovascular Diseases, School of Basic Medical Sciences, Capital Medical University, No.10 Xitoutiao, You An Men, Beijing, 100069 China

**Keywords:** Cascade initiation, Additional molar, Gene expression profiling, LincRNA, Diphyodont, Miniature pig

## Abstract

**Background:**

In diphyodont mammals, the additional molars (permanent molars) bud off from the posterior-free end of the primary dental lamina compared with successional teeth (replacement teeth) budding off from the secondary dental lamina. The diphyodont miniature pig has proved to be a valuable model for studying human molar morphogenesis. The additional molars show a sequential initiation pattern related to the specific tooth development stage of additional molars in miniature pigs during the morphogenesis of additional molars. However, the molecular mechanisms of the regulatory network of mRNAs and long non-coding RNAs during sequential formation of additional molars remain poorly characterized in diphyodont mammals. Here, we performed RNA-seq and microarray on miniature pigs at three key molar developmental stages to examine their differential gene expression profiles and potential regulatory networks during additional molar morphogenesis.

**Results:**

We have profiled the differential transcript expression and functional networks during morphogenesis of additional molars in miniature pigs. We also have identified the coding and long non-coding transcripts using Coding-Non-Coding Index (CNCI) and annotated transcripts through mapping to the porcine, Wuzhishan miniature pig, mice, cow and human genomes. Many new unannotated genes plus 450 putative long intergenic non-coding RNAs (lincRNAs) were identified. Detailed regulatory network analyses reveal that WNT and TGF-β pathways are critical in regulating sequential morphogenesis of additional molars.

**Conclusions:**

This is the first study to comprehensively analyze the spatiotemporal dynamics of coding and long non-coding transcripts during morphogenesis of additional molars in diphyodont mammals. The miniature pig serves as a large model animal to elucidate the relationship between morphogenesis and transcript level during the cascade initiation of additional molars. Our data provide fundamental knowledge and a basis for understanding the molecular mechanisms governing cascade initiation of additional molars, but also provide an important resource for developmental biology research.

**Electronic supplementary material:**

The online version of this article (doi:10.1186/s12864-017-3546-4) contains supplementary material, which is available to authorized users.

## Background

Similar to most diphyodont mammals, humans have a deciduous (primary) set of 20 teeth and a permanent set of 28-32 teeth. There are two types of permanent teeth, successional teeth, which replace primary teeth, and additional teeth (permanent molars), which develop behind the deciduous dentition without a primary predecessor. The successional teeth arise from the extension of the dental lamina on the lingual aspect of the deciduous teeth, and additional molars bud off from the posterior extension of the dental lamina. The number of additional molars varies among mammals. There are three additional molars in each quadrant in humans: the first molar (M1), second molar (M2) and third molar (M3). M3 shows more evolutionary variation in morphology, size, number (2 or 3) and location. Moreover, the process of additional molars from initiation to final eruption in humans takes a long time, from 6 (without the third molar) to 12 (with the third molar) years in different individuals.

The detailed early morphogenesis patterns of additional molars in diphyodont mammals remained unknown until very recent observations in the ferret (*Mustela putorius furo*), which has a heterodont and diphyodont dentition, very similar to humans except with only two additional molars (M1 and M2) in each quadrant. This recent work has shown that the first additional molar buds off from the posterior-free end of the primary dental lamina, in contrast to successional teeth budding off from the secondary dental lamina [1]. We recently uncovered similar patterns during early diphyodont morphogenesis in Chinese miniature pigs [2], suggesting that they are evolutionarily conserved in most diphyodont mammals. This sequential initiation in the mandible ramus related to the specific tooth development stage of additional molars in miniature pigs further indicates that dental lamina without disruption may play a key role during the morphogenesis of additional molars [2]. Our current understanding of the molecular mechanisms controlling tooth development is mostly obtained from studies in mice, which have only one set of non-replaced dentition, different from humans. For this reason, the molecular mechanisms of controlling the spatiotemporal initiation patterns of additional molars, the role of dental lamina in additional molar morphogenesis and the variation of fate determination between successional and additional teeth in diphyodont mammals remain largely unknown.

A diphyodont mammal resembling humans is required for fully understanding the morphogenesis and odontogensis of additional molars. Increasing evidence indicates that the miniature pig, a typical diphyodont mammal that also has three additional molars in each quadrant [2–4], could serve as an excellent pre-clinical alternative experimental model for studying tooth development [5, 6]. Recently, the porcine genome project was completed [7] and the genome sequence of the Wuzhishan miniature pig published [8, 9]. We have identified the stage-specific differential gene expression profiling and functional network during early morphogenesis of successional tooth development and its primary counterpart in miniature pigs [10]. These advances provide the necessary tools and expertise for dissecting developmental mechanisms between the secondary teeth and additional molars.

Here, the miniature pig serves as a large model animal to elucidate the relationship between morphogenesis and gene expression during the cascade initiation of additional molars. Using deep sequencing methods and microarray, we have profiled the differential transcript expression and functional networks during morphogenesis of additional molars in miniature pigs. We also have identified the coding and long non-coding transcripts using CNCI and annotated transcripts through mapping to the porcine, Wuzhishan miniature pig, mice, cow and human genomes. Our study offers a framework of dynamic gene expression profiling during morphogenesis of additional molars in miniature pigs and provides the molecular bases for understanding mechanisms controlling the initiation cascade of additional molars.

## Results

### Developmental patterns of additional molars

**Fig. 1:**
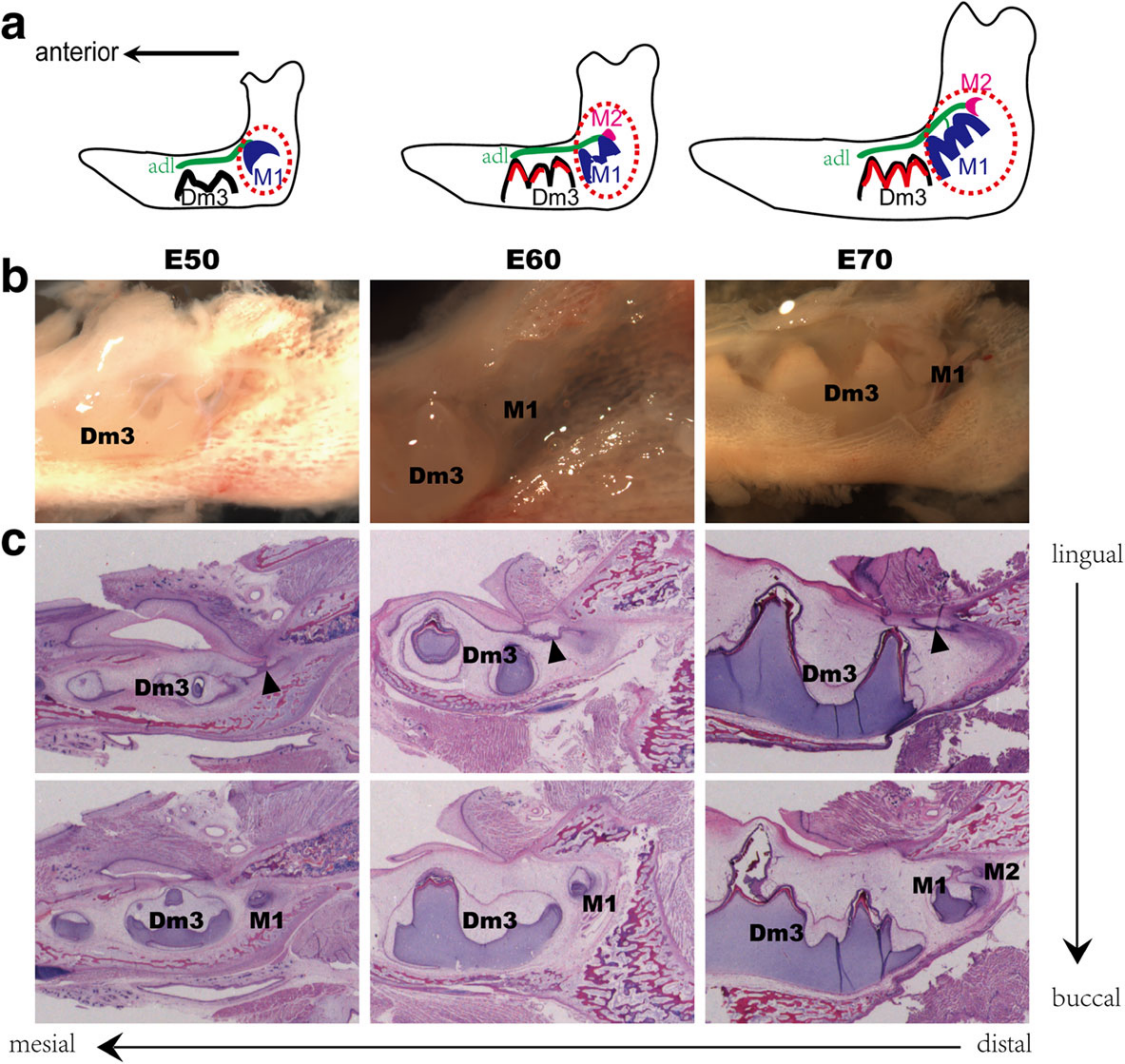
Sequential initiation pattern of mandibular additional molar during morphogenesis in the miniature pig. **a** Schematic representation of additional molar germs (dashed lines) in mandible dissected from E50, E60 and E70 miniature pig embryos for transcriptome analysis, showing a sequential initiation pattern. **b** Macro view of additional molar germ in miniature pig mandible at the different stages. **c** Serial sagittal histological sections (haematoxylin and eosin staining) show the morphogenesis of additional molars at the different stages and additional dental lamina (arrowhead) from the distal extension of the primary dental lamina. adl, additional dental lamina. Dm3, the third deciduous molar. M1, the first additional molar. M2, the second additional molar

Our previous study showed that mandibular additional molar morphogenesis in miniature pigs initiates from the distal extension of the primary dental lamina developing posterior to the third deciduous molar (designated as the additional dental lamina), which differed from replacement teeth derived from secondary dental lamina [2]. We further identified an interesting sequential initiation pattern of additional molars in the mandible ramus related to spatiotemporal morphogenesis, where M2 initiated from the posterior-free end of the dental lamina over the M1 at E60 when the M1 progressed to the late bell stage. Similarly, M3 budded off from the posterior-free end of the dental lamina over M2, which reached bell stage at PN20 (Fig. 1). We observed that the dental lamina maintained integrity without disruption during additional molar morphogenesis, suggesting spatiotemporal control of molar morphogenesis [2]. Additional molar germs from E50, E60 and E70 were chosen, respectively, as three key stages to identify the differentially expressed profiling during morphogenesis of additional molars to determine the spatiotemporal control mechanisms of cascade initiation of these molars. We chose the E50 stage corresponding to M1 at cap stage without M2 initiation and the E60 stage targeting M1 at bell stage followed by M2 initiation. We also chose E70 as a reference, when M1 progresses to late bell stage but M2 changes little. Total RNA was isolated from additional molar germs at special development stages in the mandible. The transcriptome bioinformatics pipeline using sequencing and microarray revealed the regulatory network of cascade initiation of additional molars in miniature pigs (Additional file 1: Fig. S1).

### Sequencing and functional annotation of the transcriptome

cDNA libraries were obtained from developing additional molar germs at E50, E60 and E70 in miniature pigs and normalised to increase sequencing efficiency; a total of 178,375,239 raw reads with an average length of 100 bp were generated using Illumina HiSeq 2000. About 35 GB of raw data were generated from these libraries (Table 1, Additional file 2: Table S1).

**Table 1:** Overview of transcriptome sequence assembly

	E50	E60	E70
Total clean reads	57,050,205	60,029,490	61,295,544
Total clean nucleotides	11,410,041,000	12,005,898,000	12,259,308,800
Total trinity transcripts	61,057	342,255	192,448
Total trinity components	49,282	279,928	158,626
Contig N50	1379	1159	825
Median contig length	536	352	279
Average contig	909	695	536
Total assembled bases	55,473,630	237,734,046	103,165,360
Percent GC (%)	48	51	47
Q20 percentage (%)	98	98.4	98.6

**Fig. 2:**
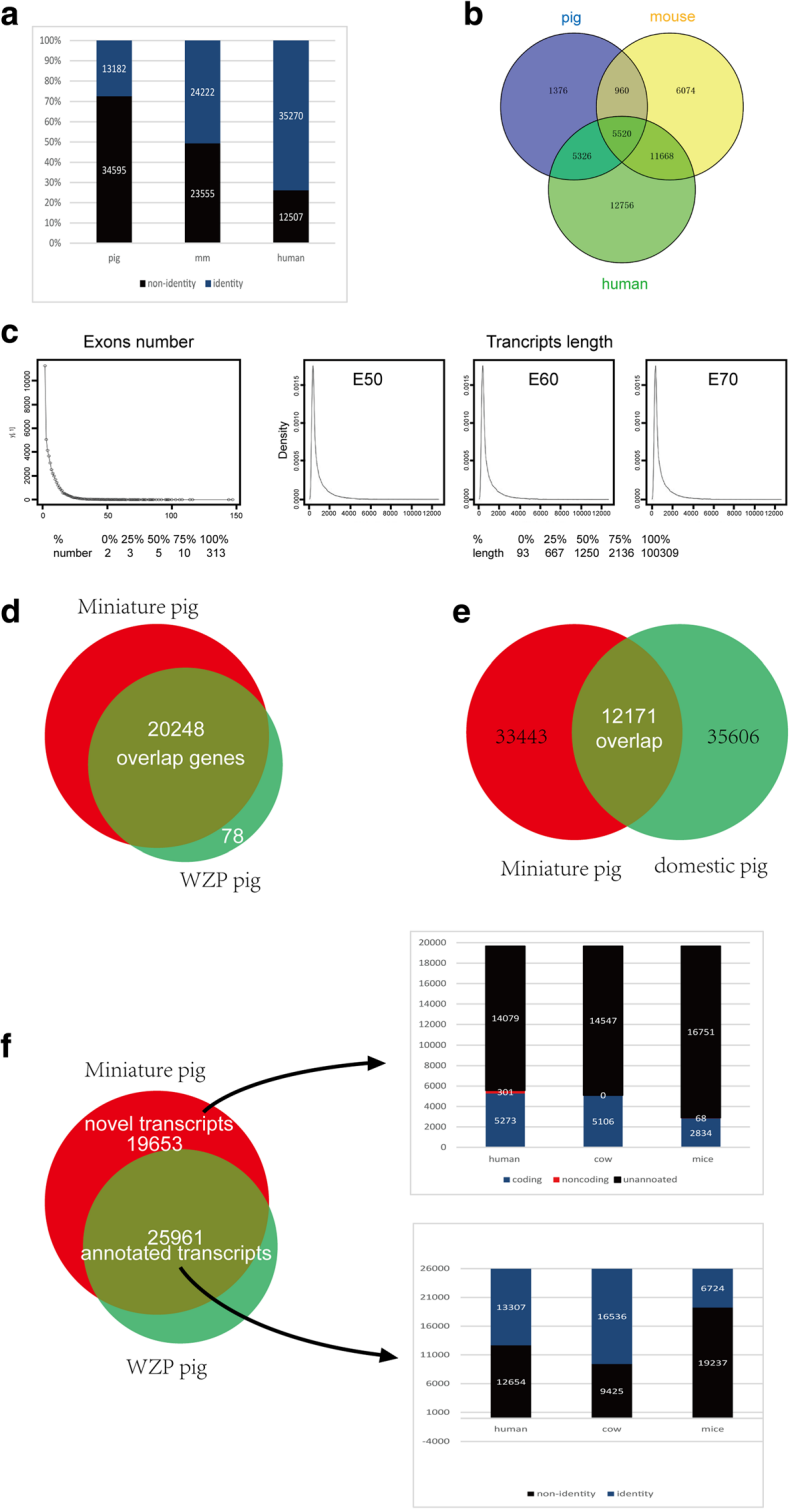
Overview of transcriptome sequence assembly. **a** The assembled 47,777 transcripts by mapping to domestic pig blasted to pig, mice and human, respectively. **b** Venn diagrams show the shared transcripts with orthologous transcripts to human, *Mus musculus* and pig genes. **c** Frequency distribution of the exon number (1 exon and 313 exons not included) and Trinity transcript length of assembled transcripts mapping to WZSP. X axis represented exon number and Y axis represented frequency of the exon number. **d** The assembled transcripts covered 20,248 in 20,326 protein-coding genes identified from the WZSP genome. **e** The identity of the transcript assembly–based domestic pig (47,777) and WZSP (45,614) reference genome. **f** The assembled 45,614 transcripts were annotated by blasting to WZSP, human, *Mus musculus* and cow

We first used the spliced read aligner TopHat (V2.04) to map all sequencing reads to domestic pig (ftp://ftp.ensembl.org/pub/release-69/fasta/sus_scrofa/dna/). Two rounds of TopHat mapping were used to maximize the splice junction information derived from all samples. Aligned reads from TopHat were assembled into the transcriptome for each sample at E50, E60 and 70 separately by Cufflinks. A total of 47,777 transcripts were identified and blasted to pig, mice and human genomes, respectively, showing that the transcriptome of the miniature pig had higher identity with human (Fig. 2a). Among these transcripts, a total of 13,182 were paralogous to pig; 24,222 were orthologous to the *Mus musculus* gene set (Ensembl GRCm38); and 36,253 were orthologous to human with good similarity. Among those blasted transcripts, 5520 transcripts were shared among pig, mouse and human (Fig. 2b). When they were blasted to the Wuzhishan pig genome (WZSP, http://gigadb.org/dataset/100031) for assessing the completeness of the assembly, the transcripts showed only few unannotated sequences mapped to WZSP (20.7%). Therefore, the sequencing reads were re-mapped onto WZSP for transcript assembly [8]. After filtering out low-quality and duplicated reads (Additional file 1: Fig. S2), the assembled transcripts from E50, E60 and E70 were merged into 979,600 transcripts by running Cuffmerge. After the transcripts with fewer than 1 exon were filtered, a total of 45,614 of 979,600 transcripts were chosen as candidates with a minimum number of 2 to a maximum of 313 exons (more than half of the transcript exon number >5 and more than half of the transcript length >1250 bp) (Fig. 2c). The assembled transcripts hit 20,248 of 20,326 protein-coding genes from the WZSP genome using intersectBed (78 of 20,326 protein-coding genes were not expressed in the tissues we obtained) with 99% overlap (Fig. 2d).

The TopHat mapping results indicated that the hits ratio mapping to WZSP was higher than that mapping to domestic pig (Table 2). Through comparison of the transcripts aligned to the assembly of domestic pig (47,777) and WZSP (45,614) reference genome, only 11,765 transcripts were found with overlap between them (8086 transcripts with functional annotation corresponding to 3322 genes), and only 12,171 transcripts aligned to the assembly of WZSP (45,614) were blasted to the domestic pig genome. A total of 25,961 of 45,614 assembled transcripts covered 99% of transcripts of WZSP, and the remaining 19,653 of 45,614 transcripts without annotation in WZSP were novel (Fig. 2e, f). The transcripts from the assembly-based WZSP genome were chosen for subsequent analysis.

**Table 2:** The hit ratio of mapping to domestic pig and Wuzhishan pig (WZSP)

Samples	domestic Pig10.2.70 (I)	WZSP (II)
E50	R1: 24.75%	R1: 28.19%
	R2: 54.67%	R2: 61.94%
E60	R1: 58.53%	R1: 63.20%
	R2: 58.01%	R2: 62.45%
E70	R1: 36.49%	R1: 40.09%
	R2: 50.97%	R2: 57.16%

The protein-coding or long non-coding transcript prediction with CNCI [11] suggested that 32,034 of 45,614 transcripts were likely to be protein-coding sequences (mRNA) and 13,580 to be long non–protein-coding transcripts. In addition to these 25,961 transcripts annotated by WZSP, the remaining 19,653 novel transcripts were blasted respectively to *Homo sapiens* in RefSeq (hg19), *Bos taurus* in (Ensembl release 72) and *Mus musculus* in RefSeq (mm9) for annotation using a homology-based method. Orthologue identification indicated that 5273 coding transcripts and 301 non-coding transcripts matched to human mRNA with best hits, 5106 coding transcripts and 0 non-coding transcripts matched to cow due to the incomplete cow database, and 2834 coding transcripts and 68 non-coding transcripts matched to mice. After filtering out redundancies and repeats, these new annotations of 19,537 transcripts were updated. A total of 45,498 transcripts with functional annotation involved in additional molar germ development were eventually obtained, of which 116 were novel transcripts without annotation in the miniature pig (Fig. 2f).

Gene expression quantification from sequencing data was performed using Cufflinks with the normalized number of fragments per kilobase of exon per million reads. Correlations of mean expression values among groups were calculated. Microarray analysis of additional molar germs from E50, E60 and E70 was also performed with Porcine Genome Arrays (Affymetrix, Inc).

Our results suggested that the high-quality assembly for miniature pig used in this study had high sequence similarity with WZSP, which offered more information for understanding the spatiotemporal expression profiling of genes during odontogenesis and morphogenesis of additional molars. These assembled high-quality transcripts were employed for subsequent analysis.

### Differentially expressed genes

After calculation and statistical analysis of the gene expression levels from the RNA-Seq data using Cufflinks, the differentially expressed genes with significance among three stages were screened by Cuffdiff (*P* < 0.05 and q < 0.05) (Additional file 3). The differentially expressed genes filtered from microarray among the three different stages were filtered using RVM f-test with MultiClassDif (*P* < 0.05, FDR < 0.05) after digital processing of the array data (Additional file 4). In total, 3109 differentially expressed genes from microarray and the top 1000 differentially expressed genes covering three stages were chosen for Pathway and GO analysis (Additional file 1: Fig. S3A). Most of the qPCR results were consistent with the expression profiles observed using microarrays or transcriptome sequencing (Additional file 1: Fig. S3B).

In the present study, 3109 of the differentially expressed genes filtered from microarray during three developmental stages were identified by RVM f-test with MultiClassDif for GO and pathways analysis (*P* < 0.05, FDR < 0.05, Additional file 4). Furthermore, 192 significant GO categories from 1213 differential expression genes were enriched (*P <* 0.01, FDR < 0.05, Additional file 4). The top 10 GO categories were gene expression, mRNA metabolic process, translational termination, RNA metabolic process, SRP-dependent cotranslational protein targeting to membrane, cellular protein metabolic process, RNA splicing, mRNA processing, translation, and small molecule metabolic process. Some GO categories, such as odontogenesis, odontogenesis of dentine-containing teeth, Wnt receptor signalling pathway, negative regulation of epithelial cell proliferation, epithelial cell differentiation and SMAD protein signal transduction, were predicted to participate in odontogenesis of additional molars and dental lamina determinants according to current knowledge and data from mammalian analyses (Additional file 1: Fig. S3A) [1, 12–17].

**Fig. 3:**
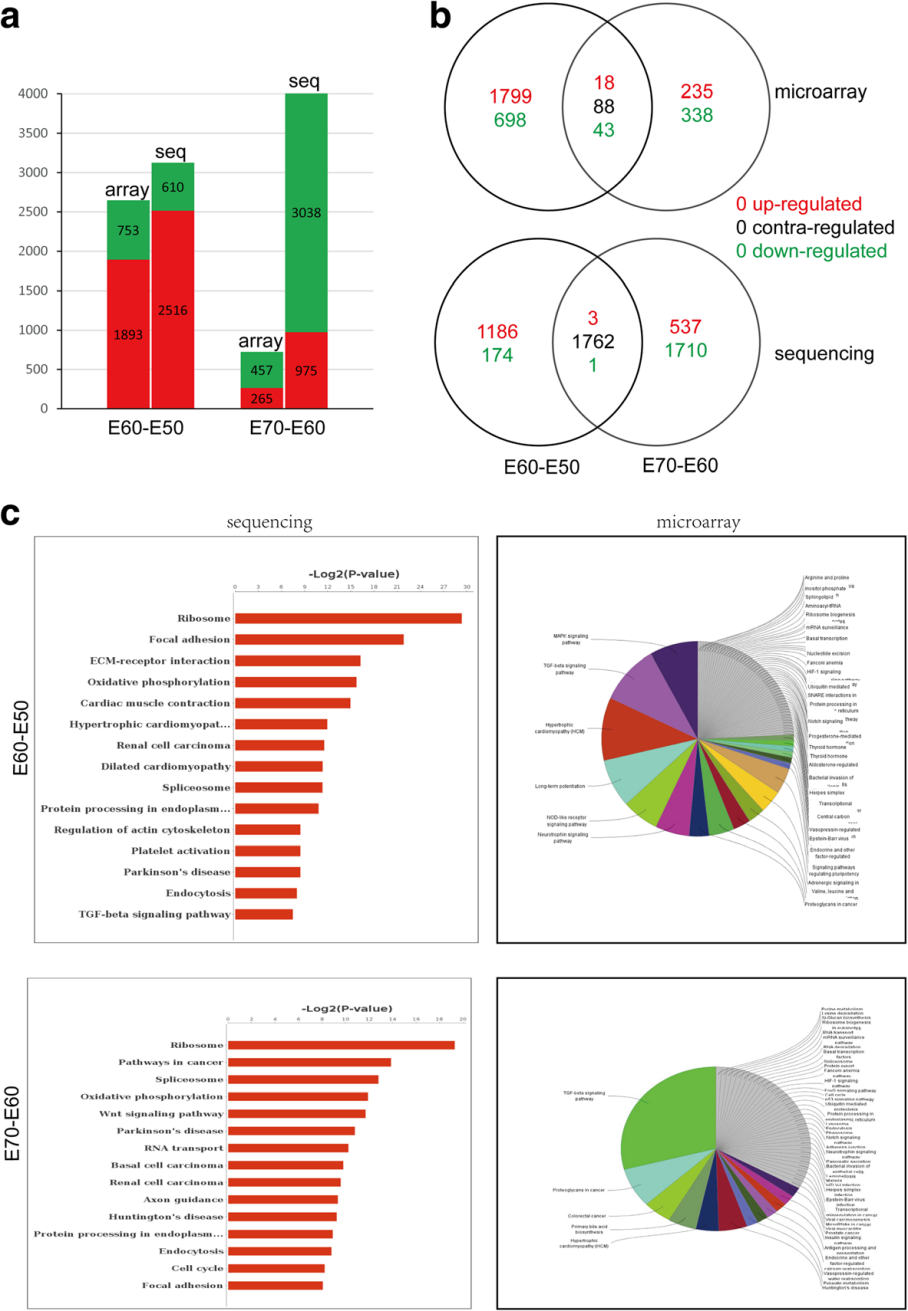
Analysis of differentially expressed genes. **a** The comparison of up-regulated and down-regulated genes from sequencing and microarray analysis during additional molar morphogenesis showing that up-regulated genes (red) are predominant at E60 versus E50, while down-regulated genes (green) are dominant at E70 versus E60. **b** Venn diagram analysis for differential expression genes from sequencing and microarray during additional molar morphogenesis showing more changes in gene expression at E60 versus E50 and relatively fewer changes between E70 and E60. **c** Pathway enrichment from microarray and sequencing between two stages showing that the WNT, TGF-β and MAPK pathways may be involved in cascade initiation of additional molars

Genes with significant differential expression at three stages were investigated. The differentially expressed genes between E60 and E50 were identified from microarray (of 2646: 1893 up-regulated genes and 753 down-regulated genes) or from sequencing (of 3126: 2516 up-regulated, 610 down-regulated). The differentially expressed genes between E70 and E60 were identified from microarray (of 722: 265 up-regulated, 457 down-regulated) or from sequencing (of 4013: 975 up-regulated, 3038 down-regulated) (Fig. 3a). Venn diagram analysis for differential expression genes during additional molar morphogenesis showed that more changes in gene expression occurred at E60 versus E50, and relatively fewer changes between E70 and E60 (Fig. 3b). The differential gene expression patterns were consistent with the fact that M1 underwent dramatic morphological changes from cap to bell stage followed by initiation of M2 and that M1 morphogenesis changed little at E70 (secretory stage) without evidence of morphological changes in M2.

These results suggested that some differentially expressed genes between E60 and E50 were involved in cascade initiation of M2 morphogenesis. The results also showed that up-regulated genes dominated M1 morphogenesis and M2 initiation. A similar pattern was obtained from microarray.

**Fig. 4:**
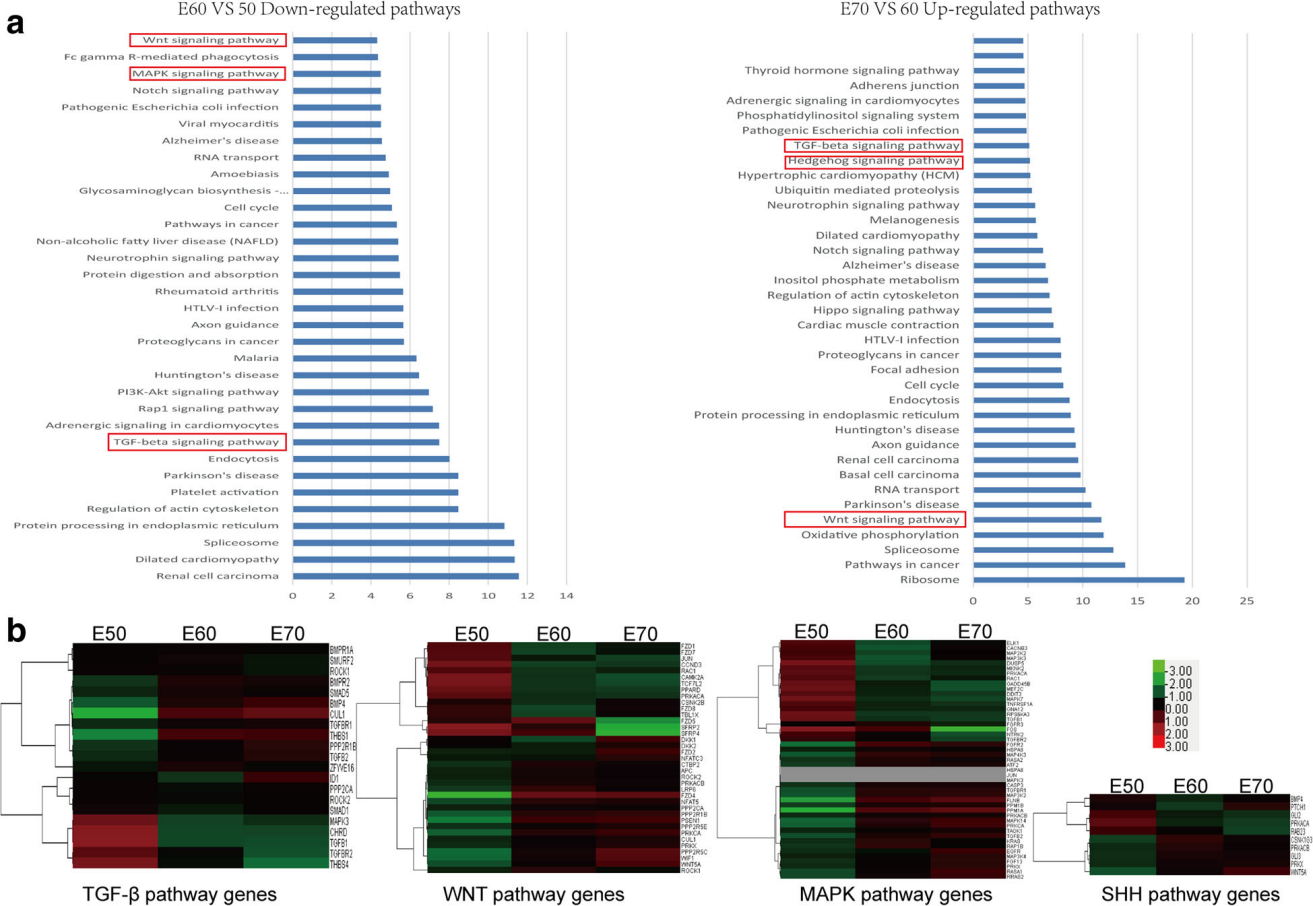
Tuning regulation of WNT, TGF-β, Hedgehog and MAPK pathways during additional molar morphogenesis from E50 to E70. **a** Down-regulated WNT, TGF-β and MAPK pathways from E50 to E60, and up-regulated WNT, TGF-β and Hedgehog (HH) pathways from E60 to E70. **b** Heatmap showing the dynamics of differential genes participating in the TGF-β, WNT, SHH and MAPK pathways. The results are based on sequences

Odontogenesis is regulated by epithelial–mesenchymal interactions mediated by conserved signalling pathways including SHH, WNT, FGF, TGF-β and BMP [17, 18]. To identify the pathways during cascade initiation of additional molars from E50 to E70, the differentially expressed genes analysed from RNA-Seq and microarrays between any two given stages were further screened, as follows. We first identified 59 significant pathways from microarrays using pathway analysis according to KEGG, Biocarta and Reatome (*P* < 0.05, FDR < 0.05, Additional file 4) for differentially expressed genes. The WNT, TGF-β and MAPK pathways were enriched, which provided additional insights into early additional molar development in miniature pigs (Fig. 3c). Our results suggested that additional molar morphogenesis is also closely related to these pathways. Moreover, we compared the differential pathways between E50 vs E60 and E60 vs E70, and pathway analysis from RNA-Seq indicated that the WNT and TGF-β pathways were down-regulated from E50 to E60 and then up-regulated from E60 to E70 (Fig. 4a), implying that additional molar morphogenesis is mediated by fine-tuning of both pathways. The genes involved in these pathways were enriched, showing dynamic changes, and were clustered for further analysis (Fig. 4b).

### Identification of signalling pathways during cascade initiation of additional molars

**Fig. 5:**
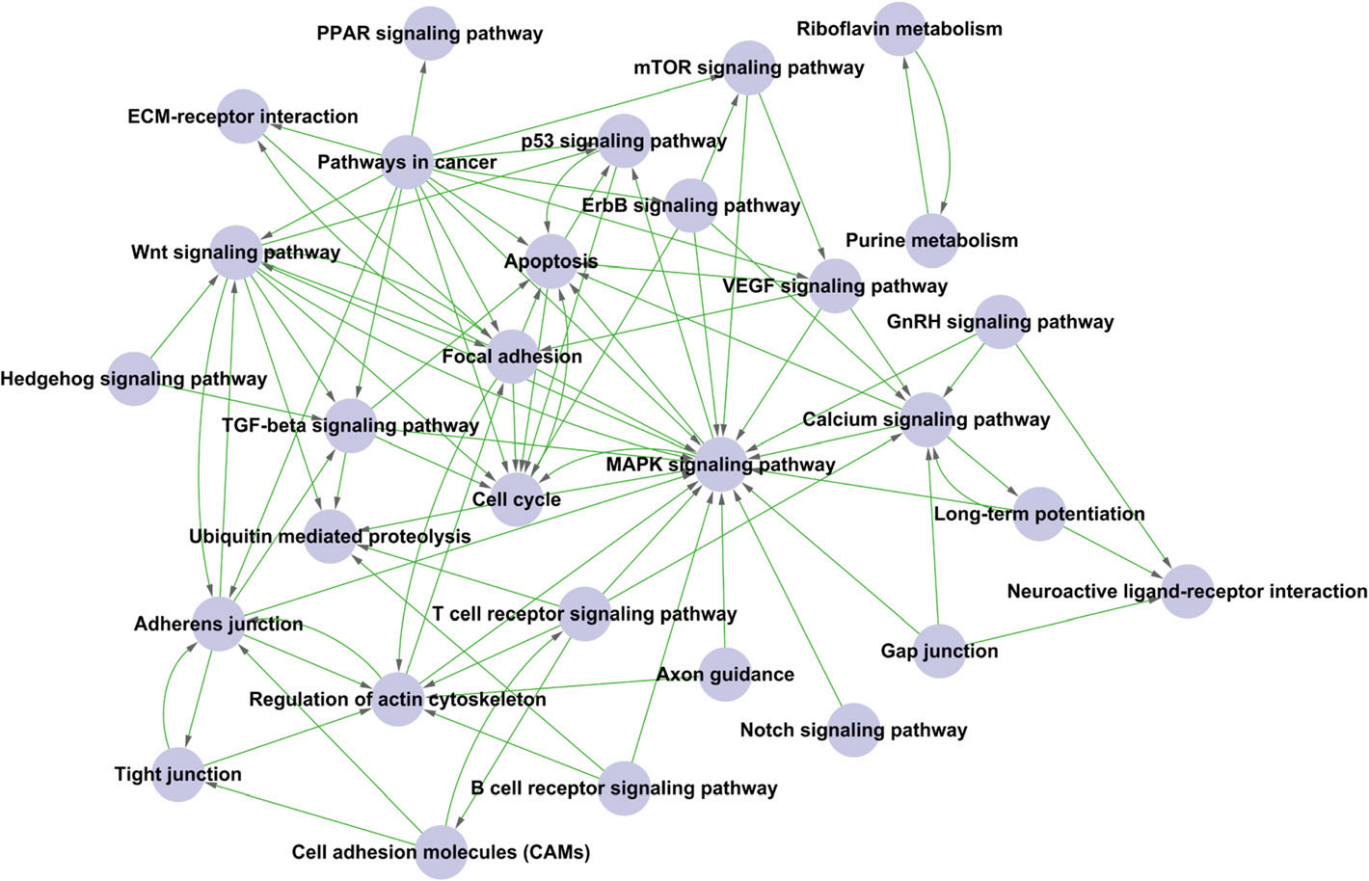
Significant pathways and clusters involving differential genes from microarray. Interactions among the significant pathways of the differentially expressed genes. Among 30 significant pathways enriched, MAPK signalling, WNT signalling, apoptosis, focal adhesion, pathways in cancer, cell cycle, p53 signalling, and TGF-β signalling show powerful target interaction capacity. Cycle nodes represent pathways, the size of nodes represents the power of the interrelation among the pathways and an arrow between two nodes represents an interaction target between pathways

Because little is known about patterning of permanent molars in diphyodont mammals, we focused on potential pathways involved in the cascade initiation for patterning of additional molars. We first selected six tendencies of gene expression from the microarray with significance from the pool of 16 potential tendencies using STC (*P <* 0.0005, Additional file 1: Fig. S4A, Fig. S5A–F) to identify which expression profiling from the differentially expressed genes was predominant during cascade initiation and morphogenesis of additional molars. The significant tendencies of gene expression can be grouped into three categories: profile 2, with constantly increased expression (Additional file 1: Fig. S5A); profile 3, with first increased then unaltered expression (Additional file 1: Fig. S5B); profile 13 (Additional file 1: Fig. S5F), which was just opposite of profile 3; profiles 4 (Additional file 1: Fig. S5C) and 7 (Additional file 1: Fig. S5E), with constantly decreased expression; and profile 5 (Additional file 1: Fig. S5D), characterized by first increased then slightly decreased expression. We then performed further pathway analysis on the most statistically significant profiles, 3 and 13 according to STC-GO analysis, suggesting that TGF-β pathway was involved in dental lamina fate (in Additional file 1: Fig. S5C, F). Profile 2, with a significant tendency to constantly increasing gene expression, was closely correlated with M1 odontogenesis by STC-GO analysis. The included genes were involved in cell adhesion, odontogenesis, odontogenesis of dentine-containing teeth, establishment or maintenance of cell polarity, TGF-β signalling pathway, cellular component movement, epithelial cell morphogenesis, epithelial-to-mesenchymal transition, regulation of epithelial cell migration, negative regulation of BMP signalling pathways, and organ morphogenesis (Additional file 1: Fig. S5B). To further define the pathways that play a central role in determining the relationship between pathways during additional molar morphogenesis, we used the differentially expressed genes in significant expression tendencies filtered from STC analysis for path-net analysis to identify significant pathways and their interactions. Thirty significant pathways involving the up-regulated and down-regulated differentially expressed genes were enriched (Fig. 5). According to target interaction capacity among pathways, some core pathways most likely related to morphogenesis of additional molars were identified, including MAPK signalling, WNT signalling, apoptosis, focal adhesion, pathways in cancer, cell cycle, p53 signalling and TGF-β signalling. These pathways consisted of 307 differentially expressed genes (Additional file 5). Combined with STC-GO analysis and the context of significant morphology changes of M1 and M2 initiation at E60, three possible pathways were enriched to contribute most likely to morphogenesis of additional molars, consisting of the TGF-β, WNT and Hedgehog signalling pathways. After the differentially expressed genes of three pathways between E60 and E50 were clustered, our results indicated that the Hedgehog and TGF-β signalling pathways were upstream of WNT signalling, implying the fine regulation of additional molar morphogenesis (Fig. 5).

**Fig. 6:**
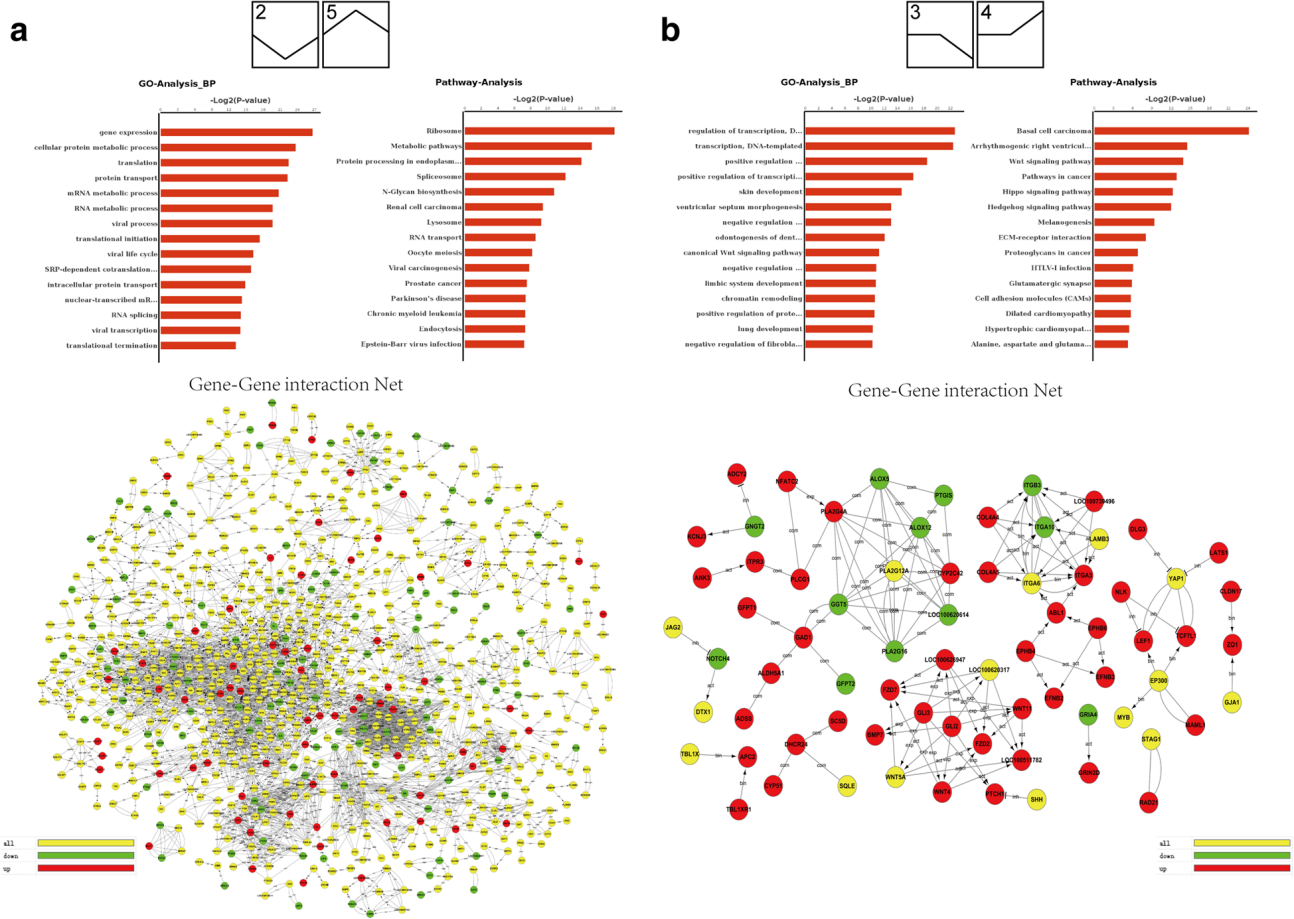
Analysis of gene expression trends from RNA-Seq. **a** The top 15 GO terms, top 15 pathways and gene-interaction net analysis from profiles 2 and 5 show that many genes and pathways are enriched from both profiles. **b** The top 15 GO terms, top 15 pathways and gene-interaction net analysis from profiles 3 and 4 suggest that both WNT and TGF-β signalling pathways are involved in both trends

Next, eight expression trends were predicted based on RNA-Seq (Additional file 1: Fig. S4B) of two profiles (profile 2 with first decreased then increased trend and profile 5 with the opposite trend) enriched with significance and many genes. After GO terms and pathways analysis, we outlined the interaction between enriched genes from significant pathways via gene–gene interaction networks (Fig. 6a). The TGF-β, WNT and Hedgehog signalling pathways were identified by GO and pathway analysis as related to both profile 3 (with an unaltered then decreased expression trend) and profile 4 (with the opposite trend), which were predicted to be related to molar morphogenesis because their trends matched the stage changes of additional molars. We also outlined the interaction between enriched pathway genes from both profiles 3 and 4 by gene–gene interaction networks (Fig. 6b). Our results provided a basis for refining candidate genes related to additional molar morphogenesis.

### Identification of a regulatory network for gene expression during cascade initiation of additional molars

**Fig. 7:**
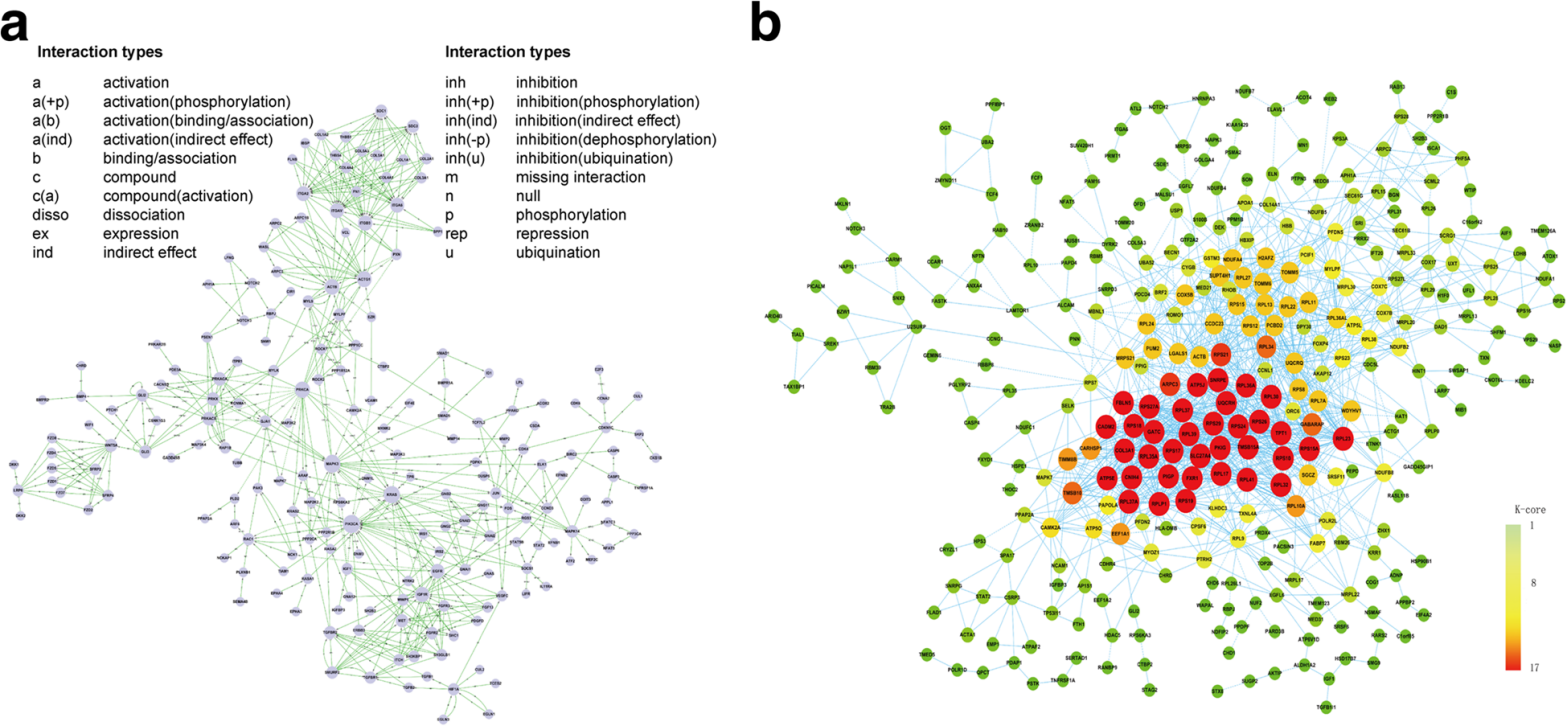
Gene–gene interaction network and Dynamic-Gene Net from microarray. Genes from significant profiles were analysed and identified by signal-net construction. **a** Gene–gene interaction network showing 23 core regulators with a higher degree, indegree, and outdegree. **b** Dynamic-Gene Net predicted 37 genes with a high degree of relevant scores as candidates for morphogenesis of additional molars (red cycle). Cycle nodes represent genes, the size of nodes represents the power of the interrelation among the genes and edges between two nodes represent interactions between genes. The more edges of a gene, the more genes connecting to it and the more central role it has within the network

To better understand the protein–protein interaction network, we screened 196 genes from microarray by STC as potential targets for morphogenesis of additional molars. We used Signal-Net and identified 23 core regulators (Fig. 7a) that had a higher degree, indegree and outdegree, indicating that they had additional interactions with other molecules in the signalling networks [19–21]. We further performed Gene co-expression network analysis to identify potential biologically meaningful genes based on a weighted connectivity score and indicators of statistical relevance among the differentially expressed genes [22, 23]. Thirty-seven predicted genes with a high degree of relevant scores were screened by Dynamic-Gene-Net as candidate targets required for morphogenesis of additional molars, which represented potential biologically relevant genes and could be used for further analysis (Fig. 7b).

### Gene expression in the SHH, WNT and TGF-β pathways during cascade initiation of additional molars

According to the above analysis, we highlighted the role of SHH, WNT and TGF-β pathways in additional molar formation. A previous study indicated that BMP4 signalling synergizes with Msx1 to activate mesenchymal odontogenic potential for tooth morphogenesis and sequential tooth formation [24]. Sox2 expression marks epithelial competence to generate teeth and is linked to the serial addition of molars with Sox2+ cells of the first molar contributing to the second and third molars in the mouse [25, 26].

**Fig. 8:**
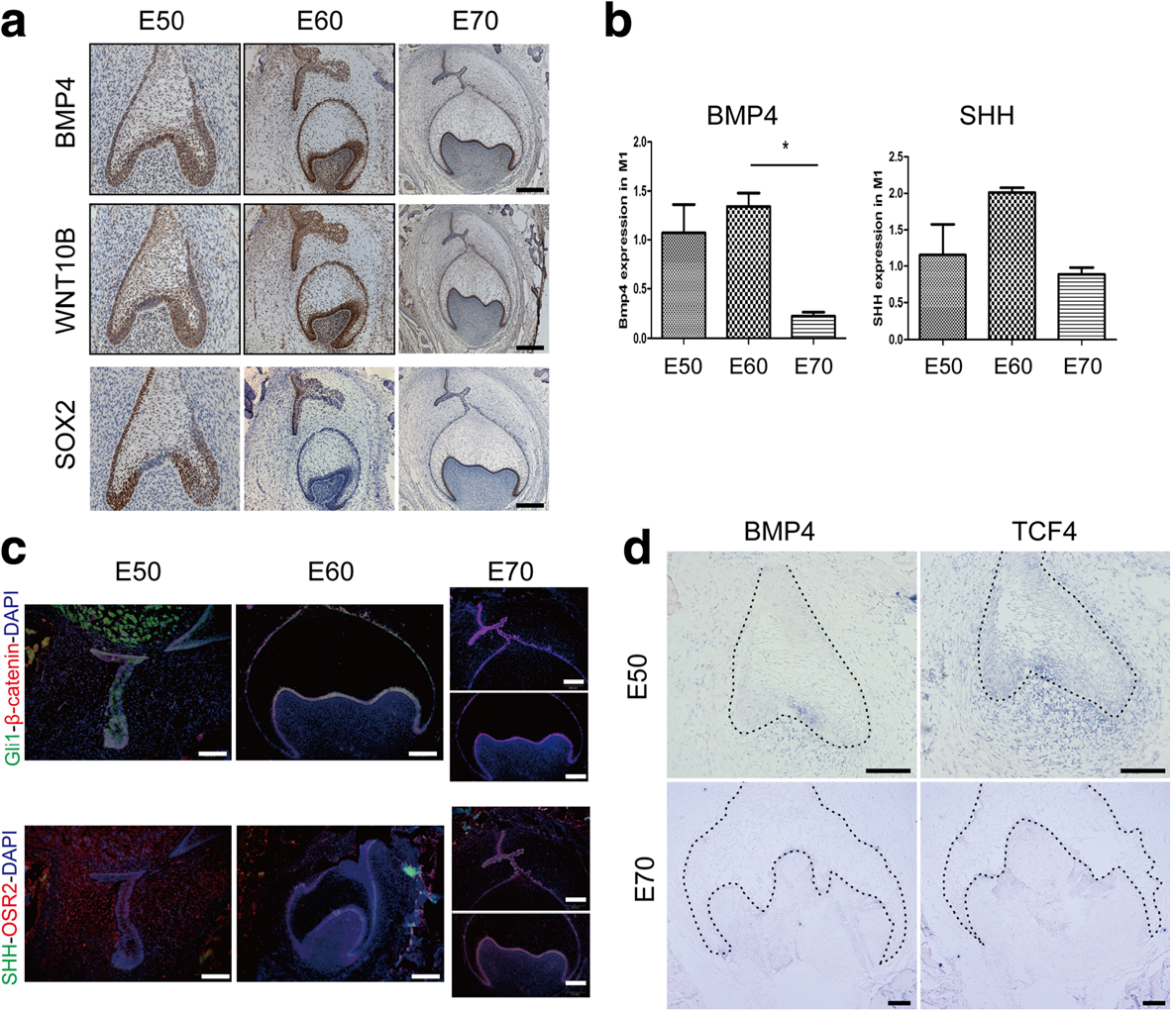
Gene expression in SHH, WNT and TGF-β pathways during cascade initiation of additional molars. **a** Immunohistochemistry of the expression of genes related to TGF-β, WNT, SHH and MAPK pathways. BMP4 expression (top panel) mainly resides in the inner enamel epithelium and outer enamel epithelium at E50; is located in the inner enamel epithelium, outer enamel epithelium, dental papilla and additional dental lamina at E60; and then is concentrated in the inner enamel epithelium at E70. WNT10b shows a similar expression pattern with BMP4 (middle panel). SOX2 expression (bottom panel), located in the inner enamel epithelium and outer enamel epithelium at E50, mainly occurs in additional dental lamina at E60 and is visible in inner enamel epithelium and additional dental lamina at E70. SOX2 expression in additional dental lamina shows an asymmetric distribution. Positive expression is visualized by DAB (brown) and counterstained with haematoxylin (blue). Lingual is to the left. Scale bars, 200 μm. **b** RT-qPCR of the expression of *BMP4* and *SHH* showing dynamic changes consistent with immunohistochemistry results. Error bars indicate s.d. three biological replicates. **c** Immunofluorescence shows the relationship among the SHH, WNT and TGF-β pathways. Immunofluorescence of Gli1 (green) and β-catenin (red) shows their co-expression in inner enamel epithelium, outer enamel epithelium and additional dental lamina (top panel); co-immunostaining of SHH (green) and OSR2 (red) in inner enamel epithelium, outer enamel epithelium, dental papilla and additional dental lamina. Lingual is to the right. Scale bars, 500 μm. **d** mRNA expression of *BMP4* and an important WNT pathway transcription factor *TCF4* detected by in situ hybridization at E50 and E70 of M1 (blue). At E50, *BMP4* is expressed in the inner enamel epithelium especially in the enamel knot and in the mesenchyme cells close to the epithelium. At E70, *BMP4* is mainly expressed in the inner enamel epithelium, similar to immunofluorescence results. *TCF4* is expressed in the inner enamel epithelium, outer enamel epithelium and dental papilla at E50 and only in dental papilla at E70. Black dashed lines mark the boundary of the tooth bud epithelium. Scale bars, 100 μm

To confirm the role of the SHH, WNT and TGF-β pathways in additional molar morphogenesis, we performed immunohistochemistry, qPCR and in situ hybridization to investigate the relationship between temporal and spatial expression distribution of some genes from SHH, WNT and TGF-β pathways and additional molar morphogenesis (Fig. 8a–d). BMP4 expression showed temporal and spatial specificity by immunohistochemistry, qPCR and in situ hybridization, and WNT10b showed a similar expression pattern with BMP4 (Fig. 8a, b, d). Sox2 expression in additional dental lamina and Osr2 expression showed asymmetric distribution in the buccal–lingual direction. Co-immunostaining indicated co-expression of Gli1/β-catenin or SHH/Osr2 in inner enamel epithelium, outer enamel epithelium and additional dental lamina (Fig. 8b, c). TCF4, a downstream effector of the WNT signalling pathway, is expressed in the inner enamel epithelium, outer enamel epithelium and dental papilla at E50 and only in dental papilla at E70 (Fig. 8d).

### Long intergenic non-coding RNA

**Fig. 9:**
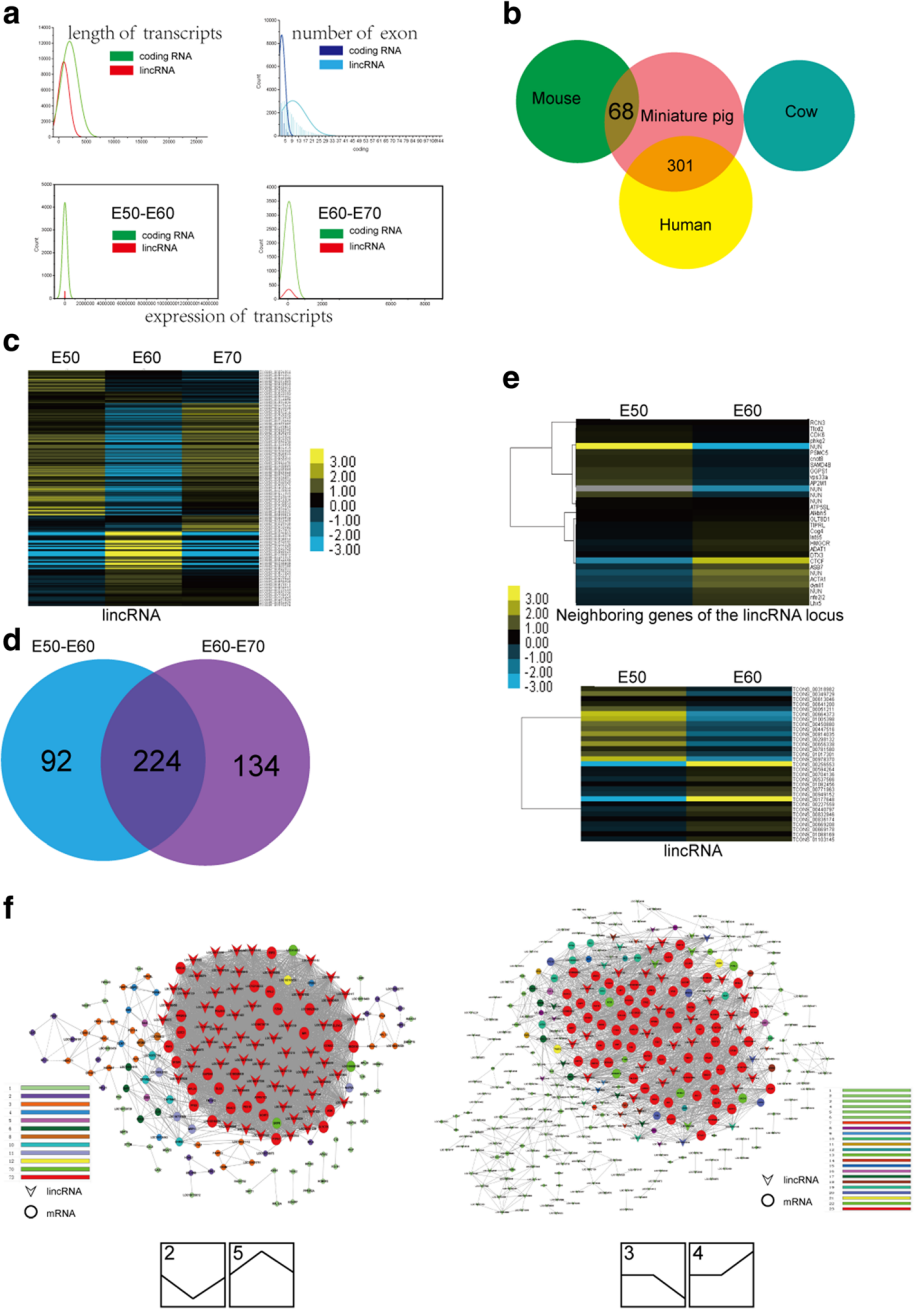
Overview of lincRNAs during additional molar morphogenesis. **a** Transcript length, exon number and expression abundance of lincRNAs were less than that of coding RNAs. **b** Identified lincRNAs showed low conservation when blasted to human, cow and *Mus musculus*. **c** Heatmap of the stage-specific expression of 450 putative lincRNAs during additional molar morphogenesis. **d** Venn diagrams of the stage-specific expression profiling of 450 differentially expressed lincRNAs show that 316 lincRNAs were identified between E50 and E60 and 358 lincRNAs between E60 and E70, and 224 lincRNAs overlapped among E50, E60 and E70. **e** Heatmap showing a negative correlation between lincRNAs and the neighbouring genes of the lincRNA locus between E50 and E60. **f** Co-Expression-Net analysis of lincRNAs and mRNAs. Left panel: genes with strong co-expression of lincRNAs (log2FC > 20 or < −20) and mRNAs (top 500 from E50 vs E60 and top 500 from E60 vs E70) from significant pathways of profiles 2 and 5. Right panel: co-expression of lincRNAs and mRNAs from significant pathways of profiles 3 and 4. Pearson coefficient > 0.999

Studies have shown that long non-coding RNAs (lncRNAs) play important regulatory roles in fundamental biological processes. These RNAs in general exhibit more cell type-, tissue- and developmental stage-specific expression profiles than mRNAs [27–31]. The contribution of lncRNAs to the additional molar morphogenesis of miniature pigs has not been reported. We obtained 13,580 candidate long intergenic non-coding RNAs (lincRNAs). Then the lincRNAs were mapped to the pig genome to obtain known and novel lincRNA transcripts. The candidate lincRNAs were further filtered by being blasted to human and mouse to exclude protein-coding genes and remove transcripts that had similarity to known proteins or protein domains. The long non–protein-coding transcripts from E50, E60 and E70 were merged using Cuffmerge to obtain 4845 lincRNA candidates. Previous studies in mammals have shown that lncRNAs are shorter, less conserved and expressed at significantly lower levels than protein-coding genes [32, 33]. We found that miniature pig lincRNAs were about half the length of protein-coding transcripts on average (e.g., 910 nt for lincRNAs vs 1945 nt for coding transcripts). Moreover, lincRNAs had fewer exons per transcript (about 2.9) than the average protein-coding gene (about 9.4). These properties are comparable to the estimated transcript length and exon number of human lncRNAs (on average, ~1 kb and 2.9 exons, respectively) [32]. The expression level of lincRNAs was lower than that of protein-coding genes, consistent with the low expression levels of their mammalian counterparts (Fig. 9a). We then aligned those lincRNAs to mouse, human (NONCODEv4 database) and cow (ftp://ftp.ensembl.org/pub/release-72/fasta/bos_taurus/ncrna/) using BLASTn. Orthologue identification indicated that only 301 non-coding transcripts matched to human mRNA with best hits, 0 non-coding transcripts matched to cow and 68 non-coding transcripts to mice (Fig. 9b), suggesting that those lincRNAs candidates were shorter, less conserved and expressed at lower levels than protein-coding genes [34, 35].

After the differentially expressed lincRNAs between two stages were further examined, we identified a final set of 450 putative lincRNAs (*P* < 0.05, q < 0.05, the average number of exons was 2.4, the average length was 857 nt, Additional file 1: Fig. S6, Additional file 6) and subsequently profiled their expression at three key stages during cascade initiation of additional molars.. The results showed those expressions exhibited temporal and spatial specificity (Fig.9 C, Additional file 1: Fig. S4C). In total, 316 differentially expressed lincRNAs were identified between E50 and E60 (41 were upregulated and 275 were downregulated); 358 differentially expressed lincRNAs were identified between E60 and E70 (289 were upregulated and 69 were downregulated), and 224 lincRNAs overlapped among E50, E60 and E70 (Fig. 9d).

Although most lncRNAs have not been functionally characterized, previous studies have shown that mammalian lncRNAs are preferentially located next to genes with developmental functions in the regulation of gene expression in either *cis* or *trans* [30]. Considering that E60 is a key stage for M2 initiation, we focused on neighbouring genes of differentially expressed lincRNAs between E50 and E60 and obtained a set of such genes for enrichment of GO. This lincRNA profile illustrated the intricate relationship between coding mRNA and lincRNA transcripts in additional molar development.

Recent studies have suggested that lncRNA expression patterns are often correlated with mRNA expression patterns to regulate the expression of neighbouring genes in *cis* or target distant transcriptional activators or repressors in *trans* [36–39]. We found that some lincRNAs are negatively correlated with neighbouring genes, implying that those lincRNAs may play a *cis* regulatory role (Fig. 9e, Additional file 7). Furthermore, we constructed networks based on trends from RNA-Seq to identify the index of the co-expression relationship between differentially expressed lincRNAs and mRNAs, suggesting that certain lincRNAs may be co-regulated in expression networks (Fig. 9f). These data may offer valuable resources for future functional studies of lincRNAs related to additional molar development.

## Discussion

In the present study, we characterized the temporal gene expression profiles of additional molars at early stages in miniature pigs. We identified key pathways participating in additional molar development and uncovered key genes and novel transcripts and lincRNAs associated with early odontogenesis and morphogenesis of additional molars. Our results showed extensive conserved homology of the transcriptome between the miniature pig and humans. In addition, the transcriptome of additional molars in miniature pigs shared similar required signals for odontogenesis with other mammals, demonstrating that the miniature pig is a much better and more biologically relevant animal model than mice for studying permanent molar development. The profiling of differentiated genes, identified pathways and key genes will further facilitate in-depth surveys of molecular mechanisms of the spatiotemporal cascade initiation of additional molars in a large diphyodont mammal. The identification of new lincRNAs will aid in advancing our understanding of their regulatory roles in this cascade initiation.

Recent studies from diphyodont mammals including ferret and swine have shown that permanent molars, being different from replacement teeth (permanent incisors, canines and premolars) initiated from secondary dental lamina, bud off from the posterior-free end of primary dental lamina [2, 10]. Similar to humans, pig permanent molars that develop behind the deciduous dentition are not replaced, forming sequentially in an anterior-to-posterior direction. We further identified a spatiotemporal pattern of additional molar cascade initiation [2]; however, the molecular mechanisms regulating sequential additional molar formation remain unclear. Studies using mouse models showed that removal of the murine M1 from the developing M2 placode in culture leads to the formation of a fourth molar (M4) [40]. An inhibitory cascade model thus has been proposed to account for sequential molar initiation in mammals. Sequential initiation of molars depends on a balance between inter-molar inhibition and mesenchymal activators. Inhibin-βA and Bmp4 are candidate mesenchymal activators whereas Sostdc1 is a candidate inter-molar inhibitor [40]. The Bmp4–Msx1 positive feedback loop has been identified as playing a critical role in driving sequential tooth formation; the Wnt–Shh–Sostdc1 negative feedback loop with a potential mechanism in which WNT signalling induces Shh to suppress the Wnt/β-catenin pathway was proposed to account for spatial tooth patterning [24, 41–43]. In the present study, the WNT and TGF-β pathways displayed associations with cascade initiation of molars. Further dissection of the functions of both pathways using the miniature pig as a model will provide key insights into the sequential regulation of the initiation of molars.

Little is known about why almost all mammals develop three molars and what limits the number of molars in most mammal species. Molar agenesis is common in humans, and the third molar is mostly affected (approximately 20% of people do not develop all third molars), followed by the second molar (0.06% in maxilla, 0.13% in mandible) and the first molar (0.07% in maxilla, 0.03% in mandible). Mutations in *MSX1*, *PAX9*, *PITX2* and *TFAP2B* have been detected in patients with molar agenesis. In the mouse, mutations in *ActRIIA/B*, *Fgf8*, *Smad2*, *Smo* and *IκBα* also present a molar-agenesis phenotype [44]. Most of these genes were identified in our study, which showed a temporal differential expression pattern during additional molar morphogenesis. Our results provide gene expression profiling that can aid in understanding molar agenesis. Moreover, the miniature pigs are convenient for performing a comparison of differentially expressed gene dynamics during replacement and additional tooth morphogenesis, which could contribute to understanding the underlying mechanism regulating permanent teeth development.

The analysis of epithelial determination implies that the dental lamina plays a key role in spatiotemporal initiation of additional molars. The stage-specific gene expression patterns suggest that up-regulated genes are predominant in E60 versus E50 while down-regulated genes dominate the morphogenesis in E70 versus E60 during early additional molar development. The combination Pathway-net and Signal-Net results suggest that sequential formation of additional molars is linked by common pathways that likely control the balance among growth, proliferation and differentiation as new dental organs initiate development. Specifically, spatiotemporal patterns of additional molars are controlled by fine-tuning the signals mediating morphogenesis and odontogenesis. Moreover, our results also imply that the WNT and TGF-β pathways strongly correlate with cascade initiation of additional molars.

A systematic analysis of lincRNAs expressed during additional molar morphogenesis has been elusive. Several studies have indicated that some lncRNAs play important roles in fundamental biological processes by a range of mechanisms, such as interacting with and modulating the activity of chromatin modification; acting as decoys in the sequestration of miRNAs, transcription factors and proteins; or serving as precursors for the generation of siRNAs [33, 45–50]. Here we provided the first systematic identification of lincRNAs during additional molar morphogenesis in miniature pigs and 450 differentiated lincRNAs. Many lincRNAs negatively correlated with genes near lincRNAs, implying that lincRNAs may play a role in regulating the silencing of neighbouring genes. Additional studies will result in a better understanding of the function and transcriptional regulation mechanism of lincRNAs during additional molar morphogenesis.

## Conclusions

Taken together, our results will not only contribute to the understanding of the molecular mechanism of additional molar developmental regulation but also provide the foundation for further exploring the regulation mechanisms of spatiotemporal variation of human permanent molars in number, size, location and eruption. The dynamic expression profiling of the identified lincRNAs will also yield novel insights into the cascade initiation of additional molars and pave the way for further explorations of the functional roles of lincRNA during porcine additional molar development.

## Methods

### Ethics statement

This study was carried out in strict accordance with the recommendations of the Regulations for the Administration of Affairs Concerning Experimental Animals (Ministry of Science and Technology, China, revised in June 2004). All procedures involving animals described in the present study were reviewed and approved by the Animal Care and Use Committee of Capital Medical University, Beijing, China (Permit Number: CMU-B20100106). All miniature pigs were placed in adjacent identical pens and given continuous access to a standard commercial feed ration and water. All surgery was performed under combination anesthesia, and all efforts were made to minimize suffering. In brief, the timed gestation of the pregnant miniature pigs was determined starting the day following insemination and verified by B-type ultrasonic inspection. The miniature pigs were anesthetized with a combination of 6 mg/kg ketamine chloride and 0.6 mg/kg xylazine, and were sacrificed by over-anesthetization after removing the fetuses by cesarean section.

### Samples collection

Pregnant Wuzhishan miniature pigs were obtained from the Institute of Animal Science, Chinese Agriculture University, Beijing, China. The staged miniature pig embryos and fetuses were obtained by cesarean section at E50, E60, and E70, and part of it subjected to histological analysis to determine the developmental stage. The additional molar germs, attached the dental lamina from the same developmental stages in mandibles from the same litter were isolated and immerged in 5–10 volumes of RNAlater solution (Ambion, USA) overnight at 4 °C, and then stored at −20 °C until needed for total RNA extraction. We distinguish three morphological developmental stages of M1: cap stage at E50, early bell stage at E60, and late bell stage at E70 respectively, verified by serial histological sections.

### Total RNA isolation

Total RNA was extracted from a pool of additional molar germs samples from same developmental stages at E50, E60 and E70 respectively using the RNeasy and RNase-Free DNase Set (QIAGEN, Germany) according to the manufacturer’s instructions. RNA integrity was verified on an Agilent Bioanalyzer 2100 (Agilent Technologies, Palo Alto, CA). RNA with RIN ≥7.0, 28S/26S ≥1.0 and OD260/280 = 1.8-2.2 was used for transcriptome sequencing, microarray and qRT-PCR analysis.

### Transcriptome sequencing and analysis

Pools of samples with a RIN score >7.0 derived from E50, E60 and E70 containing the same developmental stages were used to generate Illumina RNA-Seq libraries,which were sequenced on Illumina Hiseq 2000 at BerryGenomics Co.,Ltd (Beijing, China). Sequencing runs were performed at paired-end, 100 bp long, strand-specific reads per sample. Analysis pipeline was performed at Advanced Computing Research Laboratory (Institute of Computing Technology, Chinese Academy of Sciences, Beijing 100190, China). We used domestic pig genome (ftp://ftp.ensembl.org/pub/release-69/fasta/sus_scrofa/dna/) and Wuzhishan pig genome (WZSP, http://gigadb.org/dataset/100031) as the reference genome.

Seq-reads were aligned to the reference genome with TopHat v2.0.4. Transcripts were assembled and quantified using Cufflinks (V2.0.2). The assembled transcripts were merged by running Cuffmerge for annotation. We chose Ensembl gene sets of WZSP or pig (Sus scrofa), cow (Bos_taurus), human and mouse (mm) as reference genes for the annotation pipeline. Gene expression quantification was performed using the normalized number of fragments per kilobase of exon per million reads (FPKM) as reported in Cufflinks output. Then, GO and pathway enrichment analysis was performed for the differentially expressed genes. For lincRNAs, the transcripts (>200 bp and exon number >1) were subjected to CNCI (Advanced Computing Research Laboratory, Institute of Computing Technology, Chinese Academy of Sciences, Beijing 100190, China) [11] to get long intergenic non-coding transcript (lincRNA) candidates. The candidates from E50, E60 and E70 were merged using Cuffmerge, and aligned the short reads to WZSP, mouse, human (NONCODEv4 database hg19) and cow genome respectively for quantification and annotation using ncFANs to identify the putative lincRNAs. Raw data were transformed in FPKM (fragments per kilobase of exon per million fragments mapped) and quantiles normalized before statistical analysis. For detail of sequencing analyses, see Supplementary Methods in Additional file 8.

### Microarray analyses

According to the methods previously described [10], the GeneChip^®^ Porcine Genome Arrays (Affymetrix, Inc) were used for the expression profiling study in biological triplicates followed by manufacturer’s protocol. All microarray data have been deposited in Gene Expression Omnibus (GEO) under the accession codes GSE77006. The bioinformatics analysis was performed as described previously [10]. Next, the differential genes and functions with significant expression tendencies were predicted using STC (Series Test of Cluster) for further Path-Net, Signal-Net and Dynamic-Gene-Net analysis for Signal-Net to determine significant pathways and identify the key differentially expressed genes during early additional molars development. For detail of microarray analyses, see Supplementary Methods in Additional file 8.

### Quantitative real-time RT-PCR

The expression of a select group of genes from same RNA samples used for the microarray and sequencing analyses were analysed by real-time quantitative RT-PCR (qRT-PCR) as described previously [10]. Amplification of swine GAPDH mRNA was used as an endogenous control (primers used are listed in Table S2 in Additional file 2). Relative expression of each gene was determined using the 2^ΔΔCT^ method. Statistical analyses were performed and Pearson’s correlation coefficient was further calculated for each gene using the normalized data to quantify the consistency between microarray experiments and qRT-PCR (*p <* 0.05 and R > 0.9).

### Histochemical and immunohistochemical analysis

Sections were prepared as described previously [2]. Sections were stained with haematoxylin and eosin (H&E) for the tissue morphogenetic study. Immunohistochemistry analyses were performed following the manufacturer’s instructions. The primary antibodies used were anti-Wnt10b, anti-BMP4, anti-Gli1, anti-SHH, anti-SOX2, anti-β-catenin and anti-OSR2 (Santa Cruz). Images were taken using a microscope (Olympus BX43F) with an attached Olympus DP72 digital camera system.

### In situ hybridization

Non-radioactive in situ hybridization was performed. RT-PCR was performed using E60 embryo mandible mRNA. Degenerate primers used for miniature pig TCF4 and BMP4 are listed in Table S3 in Additional file 2. The PCR products were extracted from agarose gels.

The DNA sequencing was completed. The RNA probe was made by labelling with digoxigenin-UTP by in vitro transcription with T7 RNA polymerase according to the protocol of DIG RNA labelling Mix (Roche, USA). For the staining procedure, mandible samples were rinsed in RNAse-free PBS and fixed in 4% paraformaldehyde in PBS (pH7.5) overnight at 4 °C. The fixed tissues were decalcified in 10% EDTA-PBS for 1 week, before being embedded in paraffin. The samples were then cut into slides (4-6 μm/slide). After the rehydration through a series of ethanol (100%, 75%, 50%, and 25%), the specimens were treated with proteinase K (1 μg/ml in PBS) for 30 min at 37 °C, and then re-fixed with 4% paraformaldehyde in PBS. After rinsed with PBS, the specimens were then dehydrated with series of ethanol (25, 50, 75 and 100%), before leaving the slides to air dry for 1 h. The specimens were hybridized in hybridization buffer (containing 50% formamide, 5 x sodium citrate/sodium chloride buffer, 1% sodium dodecyl sulfate, 50 μg/ml heparin, 50 μg/ml tRNA, 1–3 μg/ml digoxigenin-labeled riboprobes) at 70 °C overnight. After washing for hours, specimens were incubated with alkaline phosphatase conjugated anti-digoxigenin Fab (Roche, USA) overnight. Positive signals were detected by incubating the specimens with NBT/BCIP substrates (Promega, USA).

## Additional files

Additional file 1: Figures S1–S6. with their legends. (PDF 5358 kb)
Additional file 2:Tables S1–S3. (DOC 45 kb)
Additional file 3: Data 1. The differentially expressed genes and GOs from sequencing. (XLSX 15911 kb)
Additional file 4: Data 2. The GOs and pathways enrichment of differentially expressed genes from microarray. (XLSX 1879 kb)
Additional file 5: Data 3. 307 differentially expressed genes from path-net analysis (*p* <0.01. (XLSX 30 kb)
Additional file 6: Data 4. 450 differentially expressed lincRNAs with significance (*P* <0.05, q <0.05). (XLSX 113 kb)
Additional file 7: Data 5. lincRNAs and neighbouring genes with negative correlation (Pearson coefficient>0.9). (XLSX 36 kb)
Additional file 8: Supplementary Methods. Including the detailed bioinformatics analysis methods not included in the main text. (DOC 69 kb)

## Abbreviations

bp: base pair
CNCI: Coding-Non-Coding Index
E: Embryonic day
FPKM: Fragments per kilobase of exon per million reads
GEO: Gene expression omnibus
lincRNA: Long intergenic non-coding RNA
M1: First molar
M2: Second mola
M3: Third molar
nt: Nucleotides
PN: Postnatal day
qRT-PCR: Quantitative real-time polymerase chain reaction
RNA-Seq: RNA sequencing
STC: Series test of cluster
WZSP: Wuzhishan pig genome
