# Supplementary material for: Transcriptome analysis of coding and long non-coding RNAs highlights the regulatory network of cascade initiation of permanent molars in miniature pigs
Source: BMC Genomics. 2017 Feb 10;18:148. doi: 10.1186/s12864-017-3546-4 (PMC5303240; doi:10.1186/s12864-017-3546-4)
Supplement: Additional file 1: Figures S1–S6. — with their legends. (PDF 5358 kb) [file 12864_2017_3546_MOESM1_ESM.pdf]

ADDITIONAL FILE1: Supplementary Figures

Transcriptome analysis of coding and long non-coding RNAs highlights the regulatory network of cascade initiation of permanent molars in miniature pigs

Fu Wang[1,2], Yang Li[1], Xiaoshan Wu[1], Min Yang[2], Wei Cong[2], Zhipeng Fan[3], Jinsong Wang[4], Chunmei Zhang[1], Jie Du[5], Songlin Wang[1,4*]

Supplementary Figures S1. The bioinformatics pipeline using sequencing and microarray methods.

Supplementary Figures S2. The filter of sequencing data from E50, E60 and E70.

Supplementary Figures S3. Overview of differential genes from microarray and sequencing.

Supplementary Figures S4. The gene expression tendencies from microarray and Sequencing during additional molar morphogenesis.

Supplementary Figures S5. STC-GO analysis from microarray.

Supplementary Figures S6. The number of exons and length of putative lincRNAs.

Supplementary Figure S1

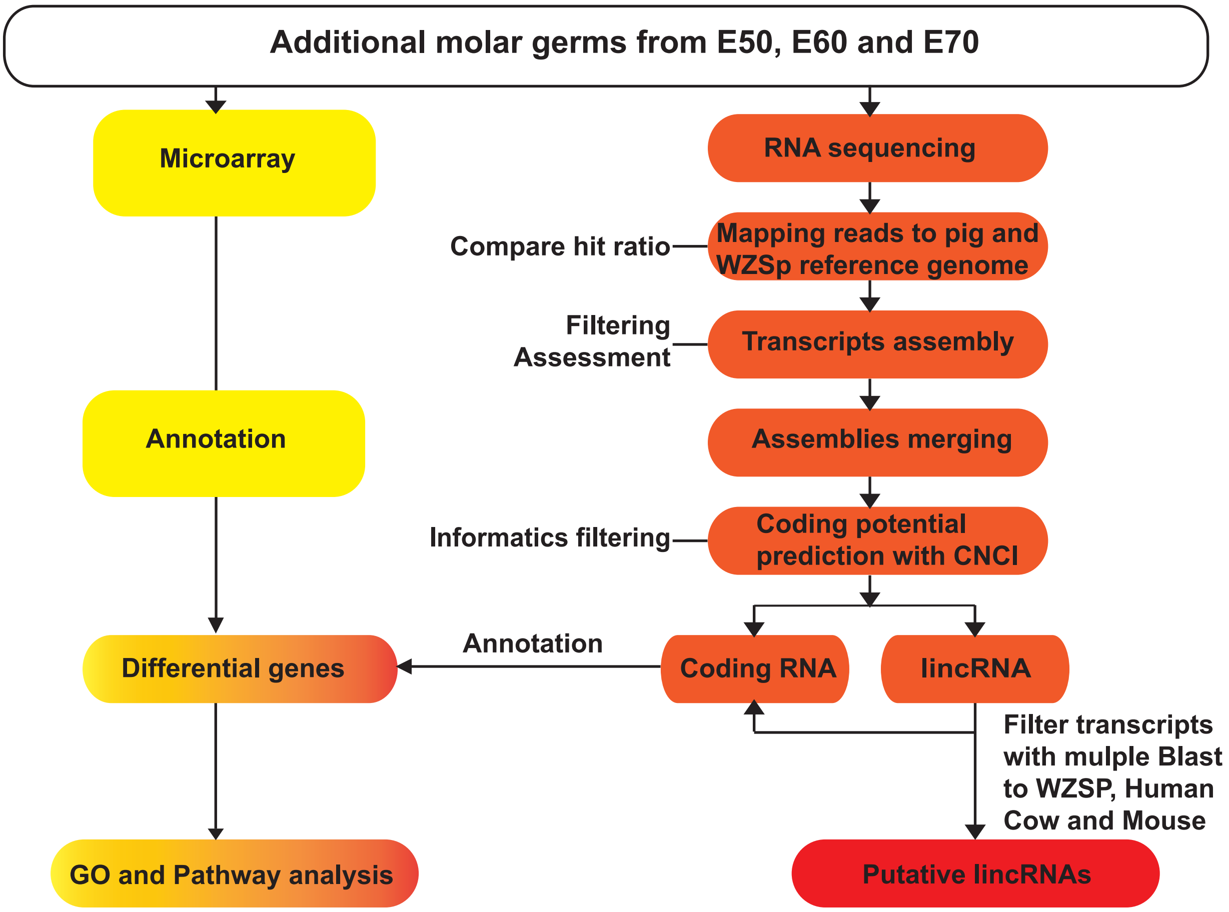

Supplementary Figure S1. The bioinformatics pipeline using sequencing and microarray methods.

Supplementary Figure S2

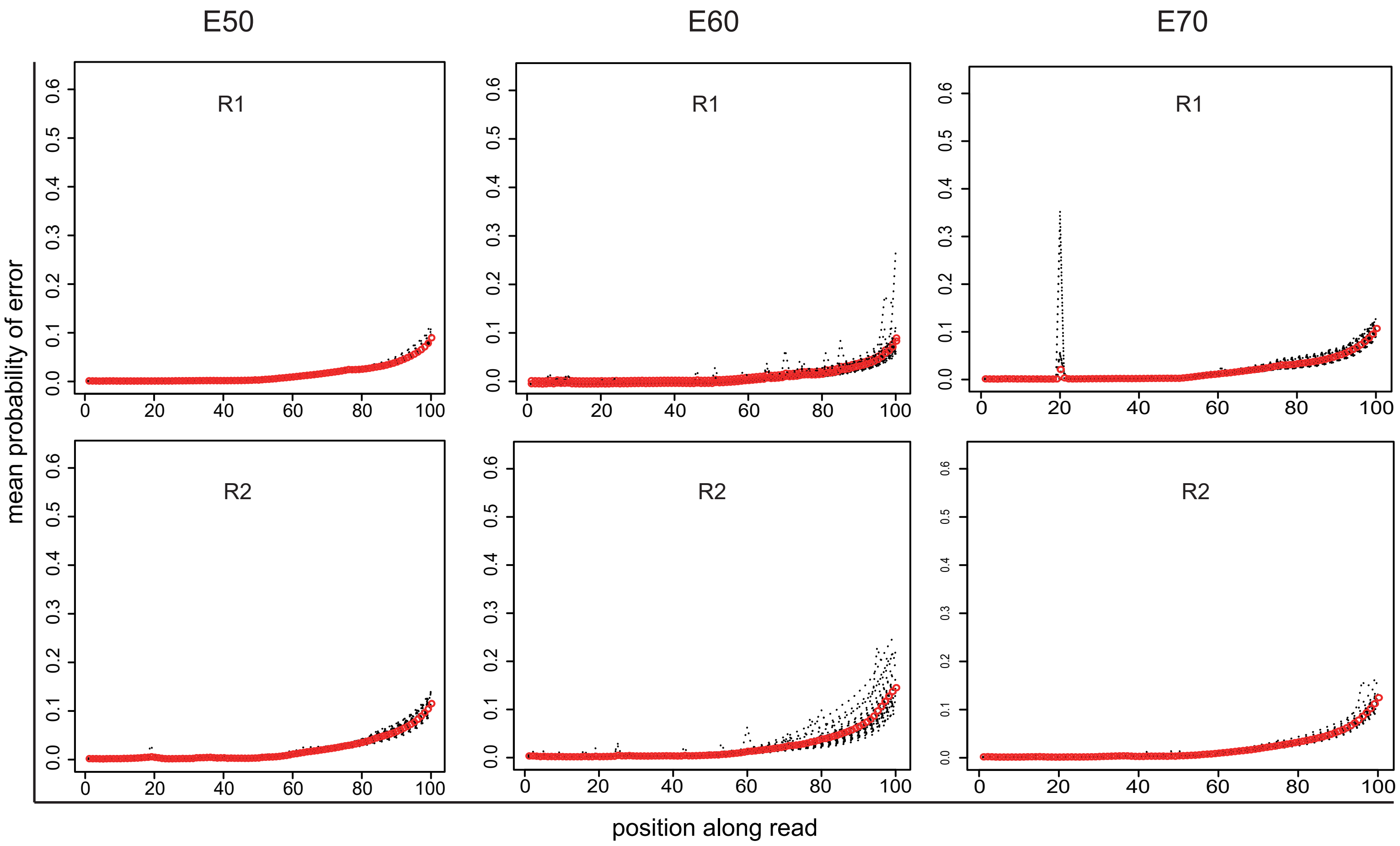

Supplementary Figure S2. The filter of sequencing data from E50, E60 and E70 showing high quality sequencing data with low probability of error (y-axis). X-axis represents positon of reads. Global average of reads in red, individual tile averages of reads in black.

Supplementary Figure S3

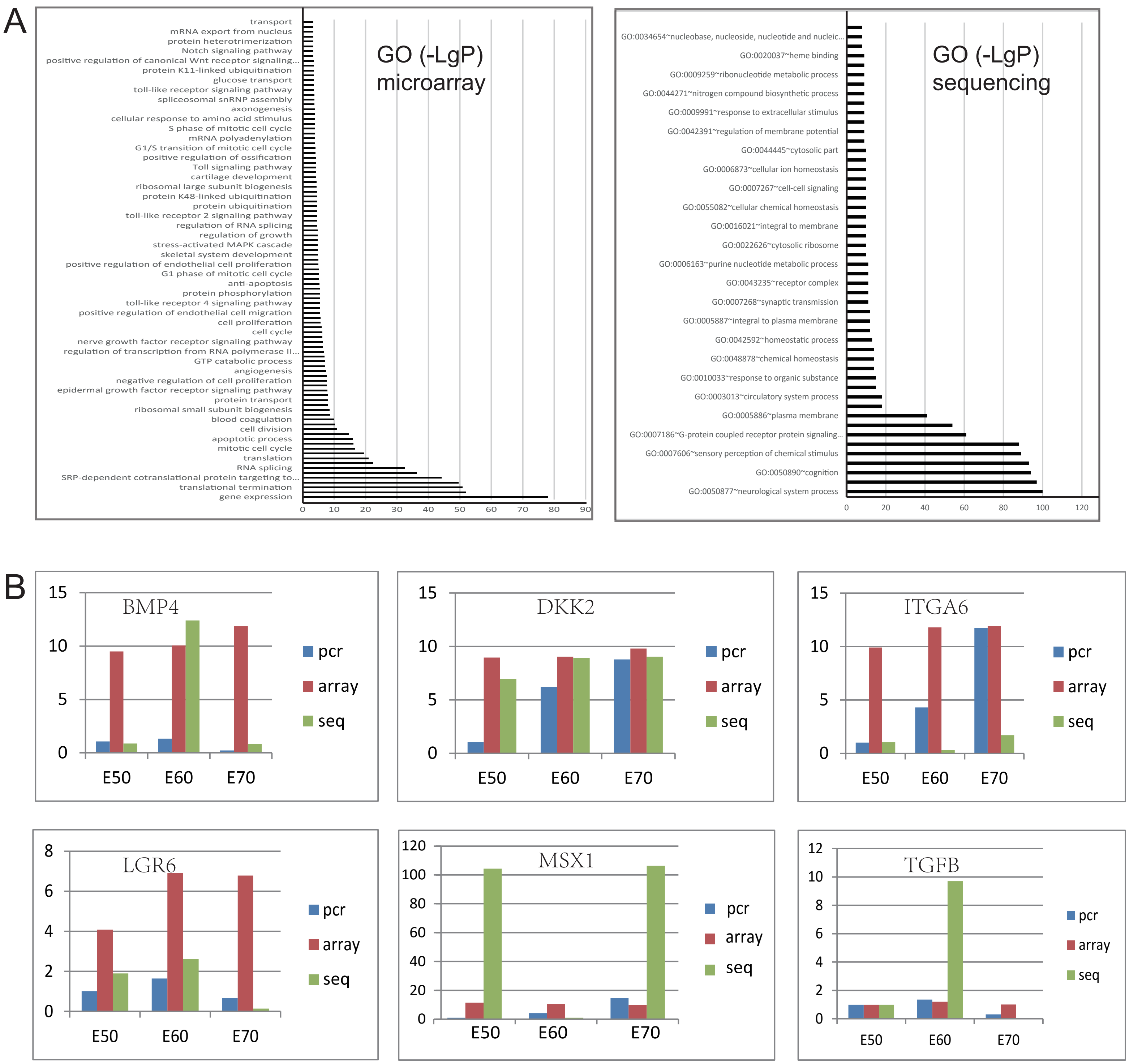

Supplementary Figure S3. Overview of differential genes from microarray and sequencing. **(A)** Top 50 GO terms of differential genes from E50 to E60 (*P*<0.05). **(B)** The genes detected by real-time RT-PCR are in agreement with the results of the normalized microarray and sequencing.

Supplementary Figure S4

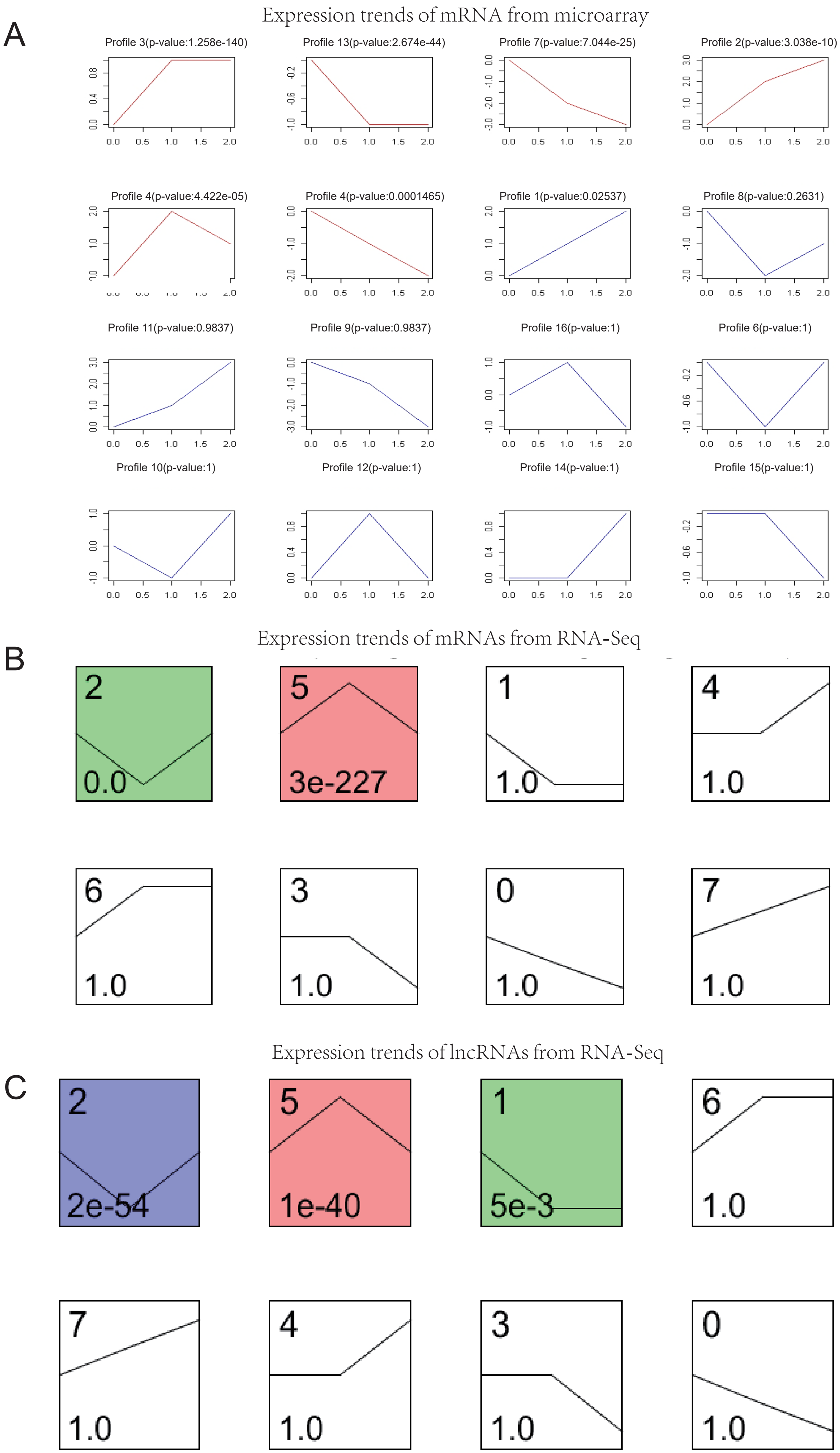

Supplementary Figure S4. The gene expression tendencies from microarray and sequencing during additional molar morphogenesis. **(A)** Total 16 potential tendencies of differentially expressed coding-RNA profiles from microarray. Each box represents a model expression profile (order based on p-value significance). Six expression patterns of genes have statistical significance ($p < 0.05$, red colored trend line). **(B)** The potential tendencies of differentially expressed coding-RNA profiles from sequencing (order based on p-value significance). The upper number in the profile box is the model profile number and the p-value is shown in bottom of box. Three significant expression tendencies are identified ($p < 0.05$, coloured boxes). **(C)** The tendencies of lncRNA expression profiles from sequencing (order based on p-value significance). The upper number in the profile box is the model profile number and the p-value is shown in bottom of box. Three significant expression tendencies are screened ($p < 0.05$, coloured boxes).

Supplementary Figure S5

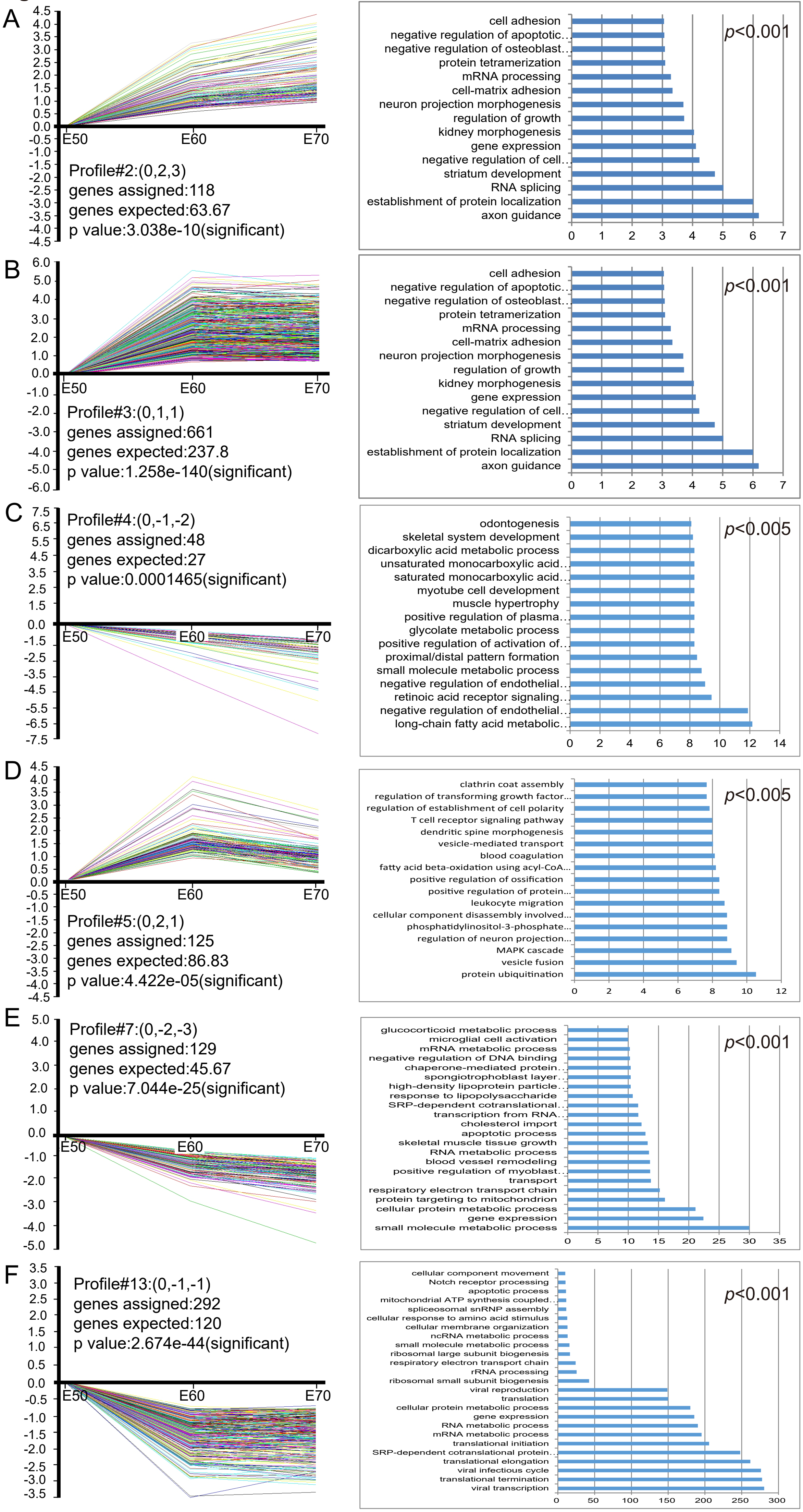

Supplementary Figure S5. STC-GO analysis from microarray. **(A-F)** the expression trends enriched from the differentially expressed genes (left panel, the horizontal axis represents stages, and the vertical axis shows the time series of gene expression levels. The value in brackets after the profile represents the variation intensity, the genes assigned represent the gene number in each profile, the genes expected represent the theoretical gene number in each profile, and the p-value indicates significance) and significant enriched GO terms (right panel). **(A)** The constantly increased expression tendency and enriched GO terms. **(B)** The tendency with first increased then unaltered expression and enriched GO terms. **(C, E)** The constantly decreased expression profile and enriched GO terms. **(D)** The tendency characterized by first increased then slightly decreased expression and enriched GO terms. **(F)** The tendency with first decreased then unaltered expression and enriched GO terms.

Supplementary Figure S6

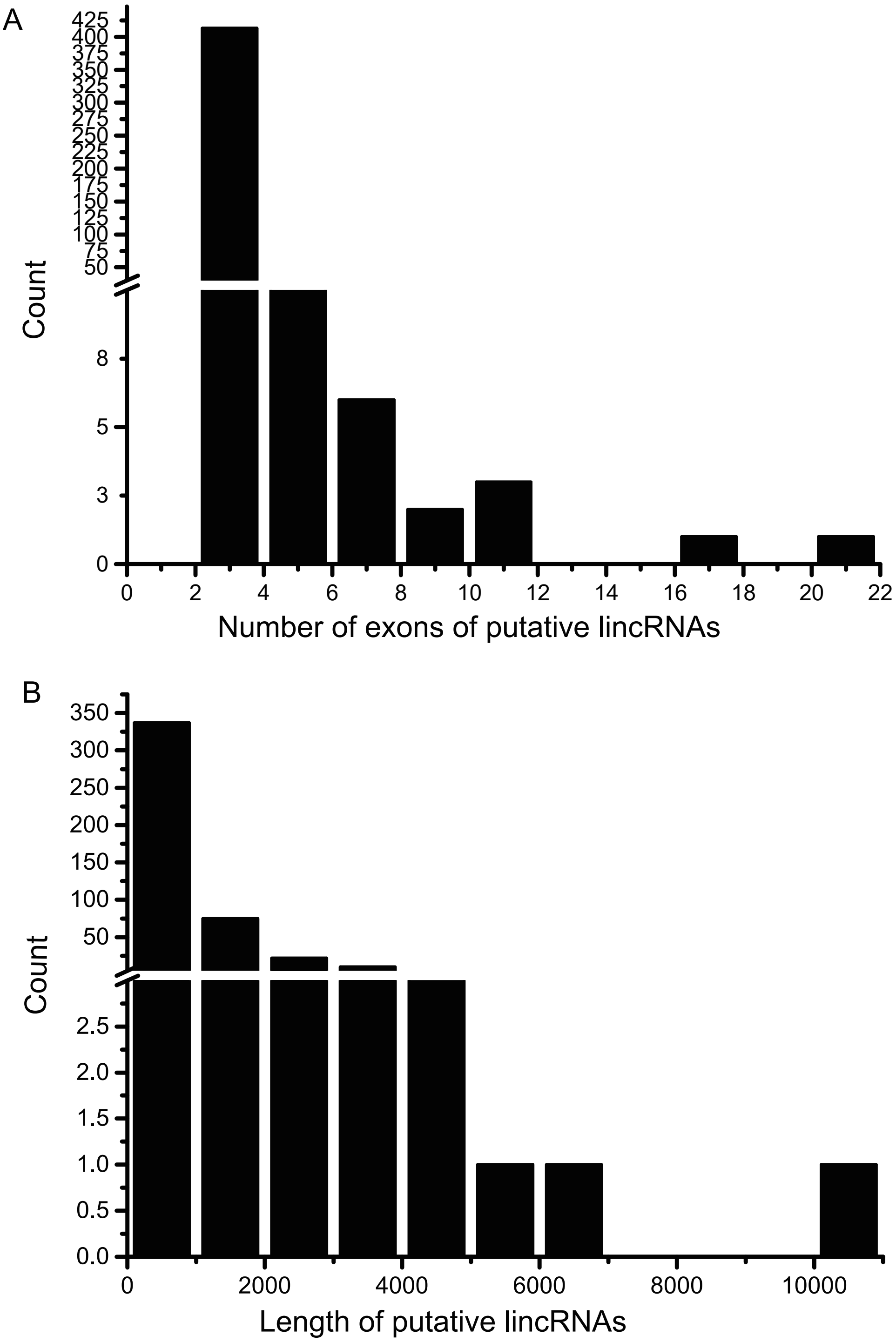

Supplementary Figures S6. The number of exons and length of putative lincRNAs. **(A)** The frequency distribution of number of exons of putative lincRNAs. **(B)** The frequency distribution of length of putative lincRNAs.
