# Supplementary material for: Transcriptome analysis of coding and long non-coding RNAs highlights the regulatory network of cascade initiation of permanent molars in miniature pigs
Source: BMC Genomics. 2017 Feb 10;18:148. doi: 10.1186/s12864-017-3546-4 (PMC5303240; doi:10.1186/s12864-017-3546-4)
Supplement: Additional file 2:Tables S1–S3. — (DOC 45 kb) [file 12864_2017_3546_MOESM2_ESM.doc]

# ADDITIONAL FILE 2: Supplementary Tables

**Transcriptome analysis of coding and long non-coding RNAs highlights the regulatory network of cascade initiation of permanent molars in miniature pigs**

Fu Wang^1,2^, Yang Li^1^, Xiaoshan Wu^1^, Min Yang^2^, Wei Cong^2^, Zhipeng Fan^3^, Jinsong Wang^4^, Chunmei Zhang^1^, Jie Du^5^, Songlin Wang^1,4*^

**Supplementary Tables S1.** Overview of the Transcriptome Sequencing.

**Supplementary Tables S2.** The Primer Sequences for real-time PCR Overview of the transcriptome sequencing.

**Supplementary Tables S3.** The Primer Sequences for PCR Prior to Probe Making for In Situ Hybridization.

**Table S1:** Overview of the Transcriptome Sequencing

| **Sample Info** | **Total clean Reads** | **Reads Length** | **Insert Size** | **Yield (bps)** |
| --- | --- | --- | --- | --- |
| E70 | 61,295,544 | 100 | 300 | 12,259,308,800 |
| E60 | 60,029,490 | 100 | 330 | 12,005,898,000 |
| E50 | 57,050,205 | 100 | 330 | 11,410,041,000 |
| Total | 178,375,239 |  |  | 35,675,247,800 |

|  |  |  |  |
| --- | --- | --- | --- |
|  |  |  |  |
|  |  |  |  |
|  |  |  |  |

**Table S2: The Primer Sequences for real-time PCR**

| mRNA name | Primer Sequences |
| --- | --- |
| GAPDH-Fn | ATTTGGCTACAGCAACAGGGT |
| GAPDH-Rn | AAGTCAGGAGATGCTCGGTGT |
| ITGB6-F | TGTATTACCTCATGGACCTCTCG |
| ITGB6-R | GCCCAGTCTAAAGTTGCTCGT |
| LGR6-Fn | TTTCCTGTGGTCTCCTTGCC |
| LGR6-Rn | CAAAGTAGCCGAAGGTCAAGG |
| DKK2/LOC100519672-F | CGTCACTTCTGGACCAAAATC |
| DKK2/LOC100519672-R | CAGTCACACCGCTGGAAGAT |
| MSX1-F | TGAGAACGGTCGGAGAAAAGA |
| MSX1-R | ACCTACCTTTGTCACGCATCTG |
| BMP4-Fn | AAGCGTAGCCCCAAGCAT |
| BMP4-Rn | CACTGAAGTCCACATAGAGCGAG |
| TGFB2-Fn | ATTTCCATCTACAACAGCACCA |
| TGFB2-Rn | CCTTGGCGTAGTATTCCTCGT |

**Table S3: The Primer Sequences for PCR Prior to Probe Making for In Situ Hybridization**

| gene name | Sense Primer | Antisense Primer |
| --- | --- | --- |
| BMP4 | GATCTCATCCGCAGAGCTTC | CTAATACGACTCACTATAGGGACTCAGTTCGGTGGGAACAC |
| TCF4 | GGCTACCCTTCCTCAAAACC | GAAACTAATACGACTCACTATAGGGACGACCCTTTGCTCCATTA |
