# Supplementary material for: Transcriptome analysis of coding and long non-coding RNAs highlights the regulatory network of cascade initiation of permanent molars in miniature pigs
Source: BMC Genomics. 2017 Feb 10;18:148. doi: 10.1186/s12864-017-3546-4 (PMC5303240; doi:10.1186/s12864-017-3546-4)
Supplement: Additional file 8: — Supplementary Methods. Including the detailed bioinformatics analysis methods not included in the main text. (DOC 69 kb) [file 12864_2017_3546_MOESM8_ESM.doc]

**ADDITIONAL FILE 14: Supplementary Methods**

**“Transcriptome analysis of coding and long non-coding RNAs highlights the regulatory network of cascade initiation of permanent molars in miniature pigs”**

by Fu Wang, Yang Li, Xiaoshan Wu, Min Yang, Wei Cong, Zhipeng Fan, Jinsong Wang, Chunmei Zhang, Jie Du, Songlin Wang

**Supplementary Methods**

**Total RNA isolation and RNA-Seq library preparation**

Total RNA was isolated from samples at specific stages using TRIZOL Reagent (Life technologies, CA, US) following the manufacturer’s instructions, and then checked for integrity using an Agilent Bioanalyzer 2100 (Agilent technologies, CA US). Total RNA was further purified using the RNeasy mini kit and RNase-Free DNase Set (QIAGEN, GmBH, Germany).

Pools of samples with RNA integrity numbers (RIN) ≥7.0 derived from E50, E60 and E70 respectively were used to generate Illumina RNA-Seq libraries. Libraries were sequenced on Illumina Hiseq 2000 at BerryGenomics Co.,Ltd ( Beijing, China) according to Illumina’s protocol. Sequencing runs were done in paired-read mode with a 100-base read length.

**Transcriptome sequencing analyses**

Analysis pipeline were performed at Advanced Computing Research Laboratory (Institute of Computing Technology, Chinese Academy of Sciences, Beijing 100190, China). We used the spliced read aligner TopHat (V2.04) to map all sequencing reads to domestic pig ([ftp://ftp.ensembl.org/pub/release-69/fasta/sus_scrofa/dna/](ftp://ftp.ensembl.org/pub/release-69/fata/sus_scrofa/dna/)), Wuzhishan pig (WZSP, http://gigadb.org/wuzhishan-inbred-pig/). Two rounds of TopHat mapping were used to maximize the splice junction information derived from all samples. In the first round, all reads were mapped with TopHat using the following parameters: multi hits = 1 and the rest set as default; in the second round of TopHat mapping, all splice junctions produced by the initial mapping were collected and fed into TopHat to re-map each sample with the following parameters: raw-juncs, no-novel-juncs, multi hits = 1. Biological replicates of mapped reads from the same sample were merged into a single BAM file to facilitate the transcript assembly and quantification.

Aligned reads from TopHat were assembled into transcriptome for each sample separately by Cufflinks (V2.0.2). Cufflinks uses a probabilistic model to simultaneously assemble and quantify the expression level of a minimal set of isoforms and provides a maximum likelihood explanation of the expression data in a given locus. Cufflinks was run with default parameters (and‘min-frags-per-transfrag = 0’) and gave out transcripts information in gtf format and expression value in FPKM (fragments per kilobase of exons per million fragments mapped).

Next, aligned reads filtered from TopHat were assembled into transcriptome by Cufflinks (V2.02) running with default parameters. The assembled transcripts were merged by running Cuffmerge. Calculation and statistical analysis of the gene expression levels from the RNA-Seq data were performed using Cuffdiff for Pathway and Go analysis.

## Protein-coding gene annotation with RNA-seq data

We principally used a homology-based method to annotate the protein-coding genes of transcripts in miniature pigs. To improve quality of protein-coding gene annotation, we chose Ensembl gene sets of WZSP or pig (*Sus scrofa*, Sscrofa10.2), cow (*Bos_taurus*, release72), human (hg19) and mouse (*Mus musculus*, GRCm38) as reference genes for the annotation pipeline. The transcripts were preferred to blast to WZSP or pig. The unannotated transcripts were to blast against cow, human and mouse. For each orthologous transcript, the aligned orthologous identity was calculated between cow, human and mouse. The reference gene with higher aligned orthologous identity to the miniature transcripts was chosen for downstream annotation. The cow’s annotation was preferred if the orthologous identity were equal, then human and mouse in turn.

## Analysis of long intergenic non-coding RNAs (linRNA) with RNA-seq data

A powerful signature tool, Coding-Non-Coding Index (CNCI), was used to identify linRNAs during additional molars development. CNCI can effectively distinguish protein-coding and non-coding sequences independent of known annotations by profiling adjoining nucleotide triplets, which is also effective for classifying incomplete transcripts and sense–antisense pairs. The transcripts from E50, E60 and E70 with low expression and a length<200bp or exon number equals one from input ﬁles were removed to avoid noise data. The remaining transcripts were merged with Cuffmerge, and then subjected to CNCI to get the linRNA candidates. The linRNA candidates overlapping with known protein-coding regions of WZSP, pig, human, cow and mouse using Cuffcompare were filtered, the remaining transcripts with length of >=200bp and exon number>1 were considered as putative linRNAs. The linRNA was functional annotated using ncFANs and then maped to WZSP resulting in known and novel linRNA transcripts. The novel transcripts were then blased to lncRNA database of mouse, human (NONCODEv4 database) and cow (ftp://ftp.ensembl.org/pub/release-72/fasta/bos_taurus/dna/) for annotation in turn. The differential expressed linRNAs with significance (p<0.05, q<0.05) between E50, E60 and E70 from Cuffdiff were identified.

## Microarray analyses

The GeneChip® Porcine Genome Arrays (Affymetrix, Inc) were used for the expression profiling study in biological triplicates followed by manufacturer’s protocol. Raw data were normalized using the MAS 5.0 algorithm of Gene Spring software 11.0 (Agilent technologies, CA, US).

The differentially expressed genes between the 3 different stages from additional molars at E50, E60 and E70 were screened with RVM f-test using MultiClassDif analyse method, then differentially expressed genes with significance were chosen according to the P value and FDR threshold for significant GO and pathway enriching [1-3].

The significant expression tendencies of genes and functions were predicted using STC (Series Test of Cluster): the raw expression values were converted into log2 ratio. The expression model profiles are related to the actual or the expected number of genes assigned to each model profile. Significant profiles have higher probability than expected by Fisher’s exact test. Go-Analysis is applied to the genes belong to certain specific tendencies. We computed P-values for the GOs of all the differential genes. Enrichment provides a measure of the significance of the function: as the enrichment increases, the corresponding function is more specific, which helps us to find those GOs with more concrete function description in the experiment [4].

Pathway analysis was used to find out the significant pathway of the differential genes according to KEGG, Biocarta and Reatome. The threshold of significance was defined by P-value and FDR [5, 6].

The Path-Net was built using Cytoscape (V3.0) according to the interaction among pathways of the KEGG database to identify which significant pathways of the differential expression genes was activated [6-8].

Signal- network were constructed using interaction database from KEGG in R. In gene networks, the number of source genes of a gene is called the indegree of the gene and the number of target genes of a gene is its outdegree. The character of genes is described by betweenness centrality measures reflecting the importance of a node in a graph relative to other nodes. The connectivity measures how correlated a gene is with all other network genes was used to identify key differentially expressed genes in the network during early additional molars development [7-9].

**Supplementary References:**

1. Wright GW, Simon RM. A random variance model for detection of differential gene expression in small microarray experiments. Bioinformatics. 2003; 19(18):2448-55.

2. Yang H, Crawford N, Lukes L, Finney R, Lancaster M, Hunter KW. Metastasis predictive signature profiles pre-exist in normal tissues. Clin Exp Metastasis. 2005; 22(7):593-603.

3. Clarke R, Ressom HW, Wang A, Xuan J, Liu MC, Gehan EA, et al. The properties of high-dimensional data spaces: implications for exploring gene and protein expression data. Nat Rev Cancer. 2008; 8(1):37-49.

4. Dupuy D, Bertin N, Hidalgo CA, Venkatesan K, Tu D, Lee D, et al. Genome-scale analysis of in vivo spatiotemporal promoter activity in Caenorhabditis elegans. Nat Biotechnol. 2007; 25(6):663-8.

5. Draghici S, Khatri P, Tarca AL, Amin K, Done A, Voichita C, et al. A systems biology approach for pathway level analysis. Genome Res. 2007; 17(10):1537-45.

6. Yi M, Horton JD, Cohen JC, Hobbs HH, Stephens RM. WholePathwayScope: a comprehensive pathway-based analysis tool for high-throughput data. BMC bioinformatics. 2006; 7:30.

7. Li C, Li H. Network-constrained regularization and variable selection for analysis of genomic data. Bioinformatics. 2008; 24(9):1175-82.

8. Pujana MA, Han JD, Starita LM, Stevens KN, Tewari M, Ahn JS, et al. Network modeling links breast cancer susceptibility and centrosome dysfunction. Nat Genet. 2007; 39(11):1338-49.

9. Ravasz E, Somera AL, Mongru DA, Oltvai ZN, Barabasi AL. Hierarchical organization of modularity in metabolic networks. Science. 2002; 297(5586):1551-5.
